# Field strains of the unicellular alga *Chlamydomonas reinhardtii* exhibit multicellular characteristics that shape their interactions

**DOI:** 10.1093/ismejo/wrag202

**Published:** 2026-07-28

**Authors:** Usha Farey Lingappa, Sunnyjoy Dupuis Borrego, Eve Sequoia Sindermann, Jude Lange Edwards, Lauren Tzu En Chiang, Jordan L Chastain, Charles Perrino, Rory J Craig, Carrie D Nicora, Samuel O Purvine, Sabeeha S Merchant

**Affiliations:** California Institute for Quantitative Biosciences, University of California Berkeley, Berkeley, CA 94720, United States; California Institute for Quantitative Biosciences, University of California Berkeley, Berkeley, CA 94720, United States; Department of Plant and Microbial Biology, University of California Berkeley, Berkeley, CA 94720, United States; California Institute for Quantitative Biosciences, University of California Berkeley, Berkeley, CA 94720, United States; California Institute for Quantitative Biosciences, University of California Berkeley, Berkeley, CA 94720, United States; California Institute for Quantitative Biosciences, University of California Berkeley, Berkeley, CA 94720, United States; Department of Plant and Microbial Biology, University of California Berkeley, Berkeley, CA 94720, United States; California Institute for Quantitative Biosciences, University of California Berkeley, Berkeley, CA 94720, United States; College of Chemistry, University of California Berkeley, Berkeley, CA 94720, United States; California Institute for Quantitative Biosciences, University of California Berkeley, Berkeley, CA 94720, United States; California Institute for Quantitative Biosciences, University of California Berkeley, Berkeley, CA 94720, United States; School of BioSciences, University of Melbourne, Parkville, VIC 3010, Australia; Earth and Biological Sciences Division, Pacific Northwest National Laboratory, Richland, WA 99352, United States; Earth and Biological Sciences Division, Pacific Northwest National Laboratory, Richland, WA 99352, United States; California Institute for Quantitative Biosciences, University of California Berkeley, Berkeley, CA 94720, United States; Department of Plant and Microbial Biology, University of California Berkeley, Berkeley, CA 94720, United States; Department of Molecular and Cell Biology, University of California Berkeley, Berkeley, CA 94720, United States; Division of Environmental Genomics and Systems Biology, Lawrence Berkeley National Laboratory, Berkeley, CA 94720, United States

**Keywords:** *Chlamydomonas reinhardtii*, algae, soil, model system, field strains, biotic interactions, multicellularity, pherophorins, biofilms

## Abstract

*Chlamydomonas reinhardtii* is a unicellular green alga long studied as a biological model system but rarely considered from the perspective of its own ecology, thus epitomizing the disconnection between reductionist biology in the laboratory and life in nature. Here, we present insights into its ecology, understood from field strains. We examined bacterial communities that coenriched with *C. reinhardtii* from the field, revealing specific associations. We then compared the biology of *C. reinhardtii* field strains to laboratory strains, illuminating strain-level heterogeneity and adaptations to life in the field vs. the laboratory. Field strains exhibited more robust photosynthesis, higher abundances of pherophorin proteins, a propensity for palmelloid formation, and high cell wall permeability. Finally, we phenotyped cocultures of *C. reinhardtii* with a coenriched bacterial partner, demonstrating how differences between field and laboratory strains manifest in biotic interactions. Although the organisms in question are classically understood as unicellular, our observations of field strains highlighted their participation in multicellular units, challenging the utility of unicellular frameworks in extending our knowledge of model organism biology in the laboratory towards understanding microbial ecology.

## Graphical Abstract

**Graphical Abstract:**
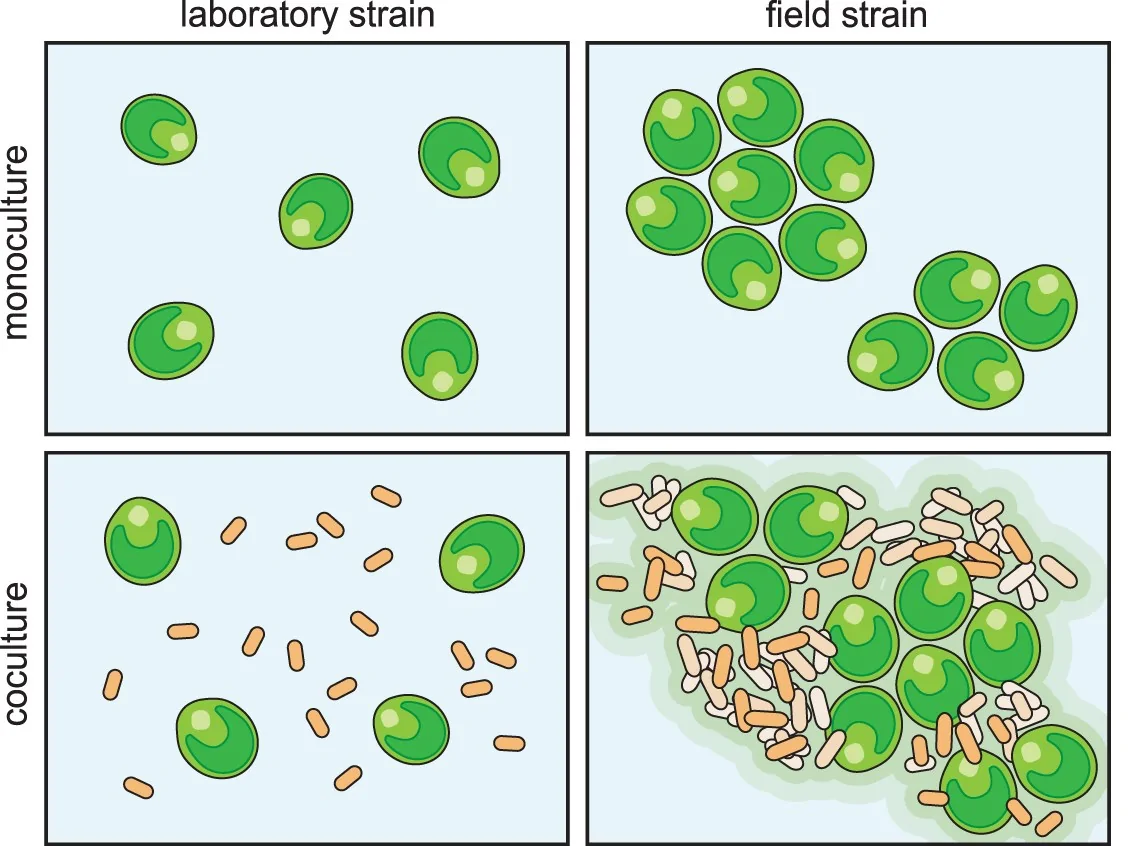

## Introduction

Ecosystem complexity obscures details of microbiological function. Therefore, much of our microbiology knowledge comes from the reductionist approach of examining isolated organisms in the laboratory. While a large fraction of microbial diversity remains recalcitrant to cultivation, a few organisms became highly studied model systems and gave us substantial mechanistic understanding of biology that would be impossible to resolve in environmental context. However, in lacking that context, what we learn from these systems may not accurately reflect how microbes operate in nature.

The unicellular green alga *Chlamydomonas reinhardtii* is a flagship model organism of the reductionist tradition [1, 2]. *C. reinhardtii* has been extensively studied for almost a century, but predominantly as a model to understand other organisms—from chloroplast biology of plants to ciliary function of animal cells—receiving little attention for its own ecology. Moreover, *C. reinhardtii* is native to soil, a notoriously complex and difficult microbial ecosystem to study [3]. Thus, *C. reinhardtii* epitomizes the disconnect between reductionist biology and ecology. We have a wealth of knowledge concerning its biology in the laboratory, with almost no understanding of how that knowledge relates to the environment.

Field strains are a bridge between the model system and the environment. We hypothesized that *C. reinhardtii* field strains recently removed from the environment would exhibit traits reflecting their ecology that were lost from laboratory strains selected over the years for manipulability in culture. Previous investigations have demonstrated extremely high genomic diversity among *C. reinhardtii* field strains [4, 5], of which an extensive investigation to generate a pangenome is underway. Here, we focused on phenotypic differences, using proteomics to capture a comprehensive view of biological function complemented with observations of metabolism, physiology, and lifecycle. We found that unlike the baseline unicellular phenotype exhibited by common laboratory strains, the field strains displayed characteristics previously described in the context of the evolution of multicellularity [6–9]. We also identified bacteria associated with *C. reinhardtii* from the field and examined cocultures with those potential symbionts; unlike cocultures with the laboratory strains that grew as individual planktonic cells, cocultures with the field strains developed multicellular physical associations resembling biofilms. Together, our results emphasize the importance of understanding that biology does not exist in isolation—it evolved and operates in the context of interactions.

## Materials and methods

### Soil sampling

We collected soil samples from Hampshire College Farm (Amherst, MA, USA) and Paradise Valley Farm (Bolinas, CA, USA). Field sites are described in Fig. S1. Samples were collected from the soil surface (≤1 cm depth) in sterile Falcon tubes using implements sterilized with 70% ethanol.

### Strains and culture conditions

We used the *C. reinhardtii* reference genome strain CC-4532 (Mets strain 2137 mt-) as our primary laboratory strain, CC-5390 (cw15 arg7-8:(ARG7) mt + [Matagne 325 background]) as an example of a cell wall-deficient laboratory strain, and AHS-32 (mt-, closely related to strain S24- [10], full haplotype information reported by Dupuis *et al.*, 2024 [11]) as a standard laboratory strain for specific coculture experiments to enable direct comparison to prior work [11]. We obtained xenic field strains from the Greater Toronto Area (GTA), Ontario, Canada (BMS-1, CB6-3, CL3-1, JG4-1, W13-1, and W13-2) from Rob Ness at the University of Toronto. Amherst and Bolinas strains were enriched from soil samples in Bold’s minimal medium under continuous light with a photon flux density (PFD) of ~100 μmol photons/m^2^/s. Enrichments were spread on agar plates, and algal colonies were screened by PCR using the primer sets described by Ford *et al.* [12]. Algal and bacterial isolates were separated from xenic cultures by serial streaking to single colonies. Algal isolates were also treated with a cocktail of antibiotics and antifungals as described by Kan *et al.* [13]. Our experimental work focused on algal strains JG4-1 and W13-2 and bacterial strain *Brevundimonas* Bl-2. This choice was driven by the fact that we had JG4-1 and Bl-2 isolated into pure cultures relatively early in this work, and the data we had generated up to that point on bacterial associates of *C. reinhardtii* implicated the genus *Brevundimonas* more frequently than any other bacterium. Thus, JG4-1 and Bl-2 represented a promising natural pairing that we could examine separately or together. We chose W13-2 as an additional algal subject to capture some of the diversity represented among the GTA set, as W13-2 was the most divergent from JG4-1 by the *YPT4* gene [12]. The Amherst and Bolinas strains had not been isolated yet at the time of this choice, and therefore are featured in the community analyses but not experimental components of this study.

Media formulations are provided in Supplemental Tables S1–S3. Algal cultures were maintained on TP or TAP agar plates. Cultures for experiments were grown in liquid TP, TAP, TAP-Fe, or HSM medium, in the dark or under continuous white light (PFD of 200 μmol photons/m^2^/s), at 24° (for trophic comparisons) or 28°C (for cocultures), shaking at 130 rpm. Unless otherwise noted, cells were collected for analysis ~48 h after inoculation. Culture specifications and cell densities for each experiment are reported in Supplemental Tables S4 and S5. Algal growth was measured by cell counts using a Z2 Coulter Particle Counter (laboratory strains) or manually in a hemocytometer (field strains). A comparison of these counting methods can be found in Fig. S2. All experiments were inoculated in three replicate flasks following two rounds of precultures grown in the same medium background as the experimental condition. Significance was determined by two-tailed Student’s *t-*test.

Bacterial cultures were grown on TY agar plates or liquid TY, TPY, or HSY, in the dark, at 30°C, shaking at 200 rpm. Precultures for coculture experiments were grown in liquid medium overnight, then washed in HSM to remove residual preculture medium. Spent medium and cell lysate were prepared as previously described [11]. Bacterial growth was measured by counting colonies grown from serial dilutions of culture spotted on TY agar plates.

### Genomic analyses

Illumina sequencing data of xenic GTA strains [12] were retrieved from the European Nucleotide Archive under accession number PRJEB56236 and imported to KBase [14]. We classified taxonomy of the raw reads using Kaiju v1.9.0 [15] with the NCBI BLAST nr + euk reference database. To generate metagenome-assembled genome (MAG) bins, we assembled the reads into contigs using MEGAHIT v1.2.9 [16], assessed assemblies with QUAST v4.4 [17], binned bacterial genomes with CONCOCT v1.1 [18] and MetaBAT2 v1.7 [19], optimized bins with DAS-Tool v1.1.2 [20], and assessed bins with CheckM v1.0.18 [21]. We then classified bins with GTDB-tk v2.3.2 [22], annotated them with RASTtk v1.073 [23], and distilled functional summaries with DRAM [24]. To identify potential gene copy number variants in W13-2 and JG4-1, we mapped reads from each strain the CC-4532 v6 assembly using bwa mem [25], computed coverage per nucleotide using the depth command in samtools v1.21 [26], and then calculated median coverage for coding sequence per gene.

Genomes of bacterial isolates co-enriched with GTA strains were sequenced and assembled by SeqCenter and deposited in JGI GOLD under organism ID numbers Go0702081, Go0715798, Go0715799, Go0715800, Go0715801, and Go0715802.

We analyzed the microbial communities in Amherst and Bolinas xenic algal strains and source soil samples by 18S and 16S rRNA amplicon sequencing. DNA was extracted using the Qiagen PowerSoil Pro kit. Amplicons were prepared and sequenced by CALeDNA [27]. Data were processed and deposited using the eDNAexplorer platform [28]. Further taxonomy assignments were done using SILVA [29], and 18S tree was generated in Geneious, aligned with MUSCLE [30] and built with PhyML [31].

### Proteomic analyses

Samples were prepared and analyzed by TMT-labeled peptide mass spectrometry as previously described [32]. Raw data were deposited in the MassIVE repository under accession number MSV000100518.

Proteomic data are presented here as protein abundance values and z-scores of mean abundance values across all strains and conditions. The abundance values are derived from reporter ion abundances extracted from MS2 spectra via MASIC [33], grouped by unique peptide sequence and parsimony filtered gene ID, summed across within-plex fractions, log_2_ transformed, and mean central tendency normalized. Global analyses included all proteins detected above the LOQ in >2 replicates of all conditions. Additional proteins detected in all samples of individual strains but not detected in other strains were included in specific analyses of cell wall and ribosomal proteins. Pherophorins were identified by Pfam domain PF12499; other proteins of interest were identified by annotations in the *C. reinhardtii* v6.1 genome. Analyses were conducted using the R packages PCAtools v2.14.0, stats v4.3.2, and clusterProfiler v4.10.1, and visualized using ggplot2 v3.5.1 and pheatmap v1.0.12.

### Chlorophyll fluorometry

We measured the maximum quantum yield of PSII (*F_v_/F_m_*, where *F_m_* is the maximum chlorophyll fluorescence following a saturating light pulse and *F_v_* is the difference between *F_m_* and *F_0_*, the initial fluorescence of dark-adapted cells) using a Walz IMAG-MAX/L MAXI Imaging PAM, as previously described [32].

### O_2_ production and consumption

We measured O_2_ production and consumption rates using a Hansatech Oxygraph Clark-type electrode. Respiratory O_2_ consumption was determined as the O_2_ consumption rate measured in the dark. Photosynthetic O_2_ production was determined as the O_2_ production rate measured in the light minus the respiratory rate measured in the dark, as previously described [34].

### Inductively coupled plasma mass spectrometry

Cells were collected by centrifugation at 2500 × *g* for 3 min, washed in 1 mM EDTA pH 8, and digested in 70% nitric acid at 60°C overnight. Biomass elemental profiles were measured with an Agilent 8900 inductively coupled plasma mass spectrometer (ICPMS), as previously described [35].

### Cell wall permeability

Cultures were treated with Triton X-100 at a final concentration of 0.1%, mixed for 4 min, and then collected by centrifugation at 10 000 × *g* for 2 min. Absorbance at 680 nm was measured with a plate reader on starting cultures, supernatants after detergent treatment, and supernatants from control cultures not treated with detergent. Data are presented as the percent of background subtracted absorbance of the starting culture released into the supernatant by detergent treatment.

### Total organic carbon

Cells were collected by centrifugation at 20 000 × g for 2 min and digested in 6 N HCl at 65°C overnight. Supernatants and digested pellets were diluted in milliQ water to obtain total organic carbon (TOC) concentrations within the linear range (0.5–25 μg/ml). Supernatant samples were supplemented with HCl to the same final concentration as digest samples. The nonpurgeable TOC content was determined with a Shimadzu TOC-L total organic carbon analyzer.

### Microscopy

For light microscopy, cultures were immobilized in a solution of 0.025% I_2_ and imaged with a Zeiss Axio Lab A1 microscope. For fluorescence microscopy, cultures were fixed in 4% paraformaldehyde in the dark for 1 h at room temperature or 4°C overnight, washed with 1× PBS pH 7, and stored in 1:1 PBS:ethanol at −20°C. Fixed cultures were spotted on 0.22 μm isopore polycarbonate filters, washed with sterile water to remove residual salts, and mounted on slides with 5 μg/ml DAPI in Citifluor antifade mounting medium buffered with PBS. Filters were imaged using a Zeiss Axio Imager M2 microscope with DAPI (excitation at 350 nm) and CY5 (excitation 620 nm) filter sets.

## Results and discussion

### Soil algae associate with specific bacteria

Interactions between *C. reinhardtii* and bacteria are often studied as model autotroph–heterotroph interactions [36]. We hypothesized that field strains and the bacterial partners associated with them in the environment might engage in interactions distinct from those captured by laboratory strains and model partners. Therefore, we sought to identify the bacteria that coenriched with *C. reinhardtii* from the field.

We obtained a set of xenic *C. reinhardtii* field strains from soil of the GTA, Ontario, Canada (strains BMS-1, CB6-3, CL3-1, JG4-1, W13-1, and W13-2, initially described by Ford *et al.* [12]). Each strain was propagated from a single algal colony and thus contained a clonal population of the alga, but still retained other organisms that coenriched with the alga from the field. We conducted an isolation campaign to separate the algae and their associated bacteria into pure cultures and recovered bacterial isolates of the genera *Brevundimonas, Caulobacter, Hydrogenophaga, Microbacterium, Pseudomonas*, and *Variovorax*.

**Figure 1:**
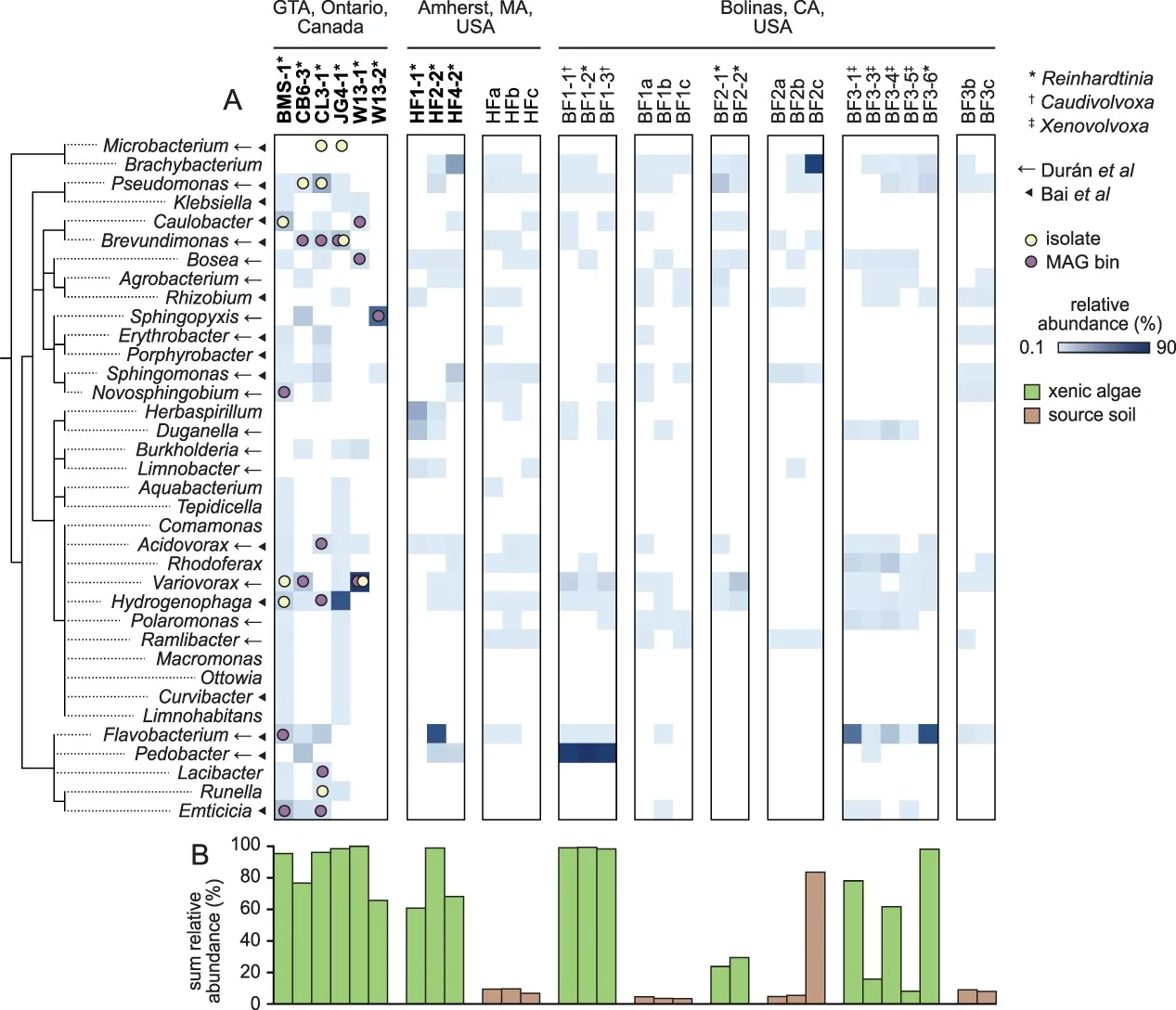
Bacterial associates of *C. reinhardtii* from the field. (A) Relative abundance of recurring *C. reinhardtii* associates (bacterial genera identified in >1 xenic *C. reinhardtii* strain, clustered by taxonomy) in *C. reinhardtii* strains from GTA, Ontario, Canada; *C. reinhardtii* strains and source soil communities from Amherst, MA, USA; and other Chlamydomonads and source soil communities from Bolinas, CA, USA. Bolded sample names indicate *C. reinhardtii* strains; symbols indicate broader algal taxonomic groups; arrows indicate bacterial genera identified by Durán and Flores-Uribe *et al* [37] and Bai *et al* [38]; circles indicate bacterial isolates and MAGs. (B) Fraction of all bacterial reads from each sample represented by the taxa presented in (A).

For a cultivation-independent window into their microbiota, we examined whole genome sequencing data generated from the GTA algae in their xenic state [12]. In some cases, we recovered more reads assigned to bacteria than to the alga itself (Fig. S3). We analyzed the bacterial community composition represented in these data and were struck by the substantial recurrence of the same bacterial genera in different algal strains from different soil samples (Fig. 1A). Genomic comparison of our *Brevundimonas* isolates and MAGs from the xenic DNA data demonstrated that the Brevundimonads associated with each alga were not identical, and therefore, their recurrence was not an artifact of cross contamination (Fig. S4). Given the overwhelming complexity of soil microbial communities, such recurrence suggested specific associations.

To compare algal-associated communities to their source soil communities, we enriched for additional strains from Amherst, MA, USA and Bolinas, CA, USA (Fig. S1). We found abundant *C. reinhardtii* in Amherst and other Chlamydomonads but not *reinhardtii* in Bolinas (Fig. S5). We sequenced 16S rRNA amplicon libraries from xenic algal cultures and source soil samples for microbial community analyses. We again observed a high overlap in the bacteria detected in the xenic cultures, not only among *C. reinhardtii* strains, but also with the Bolinas algae, suggesting similarities in the phycosphere niche supported by different soil Chlamydomonads. The genera repeatedly coenriched with *C. reinhardtii* collectively represented most of the bacteria in the xenic cultures but only a small fraction in the source soil (Fig. 1B). The gene content of all the MAGs we recovered of these bacteria demonstrated heterotrophy and oxygen respiration capacity (Fig. S6), as expected from direct symbionts of oxygenic primary producers, which does not reflect the metabolic diversity of bulk soil communities.

We interpreted the bacteria that repeatedly coenriched with *C. reinhardtii* but were not dominant members of source soil communities as meaningful symbiotic associations. This interpretation was strengthened by recent work from other groups. Durán and Flores-Uribe *et al.* [37] seeded soil microbial communities with *C. reinhardtii* cultures and identified taxa that were reproducibly enriched in the presence of the algae. Bai *et al.* [38] published a similar study with freshwater microbial communities. Both of those analyses highlighted many of the same taxa as ours. Of the 9 bacterial genera that we found associated with >3 *C. reinhardtii* field strains—*Acidovorax, Bosea, Brevundimonas, Caulobacter, Flavobacterium, Hydrogenophaga, Pseudomonas, Sphingomonas*, and *Variovorax*—Durán and Flores-Uribe *et al.* identified 7 and Bai *et al.* identified 8 (Fig. 1A).

### Growth of *C. reinhardtii* field strains under different trophic conditions

**Figure 2:**
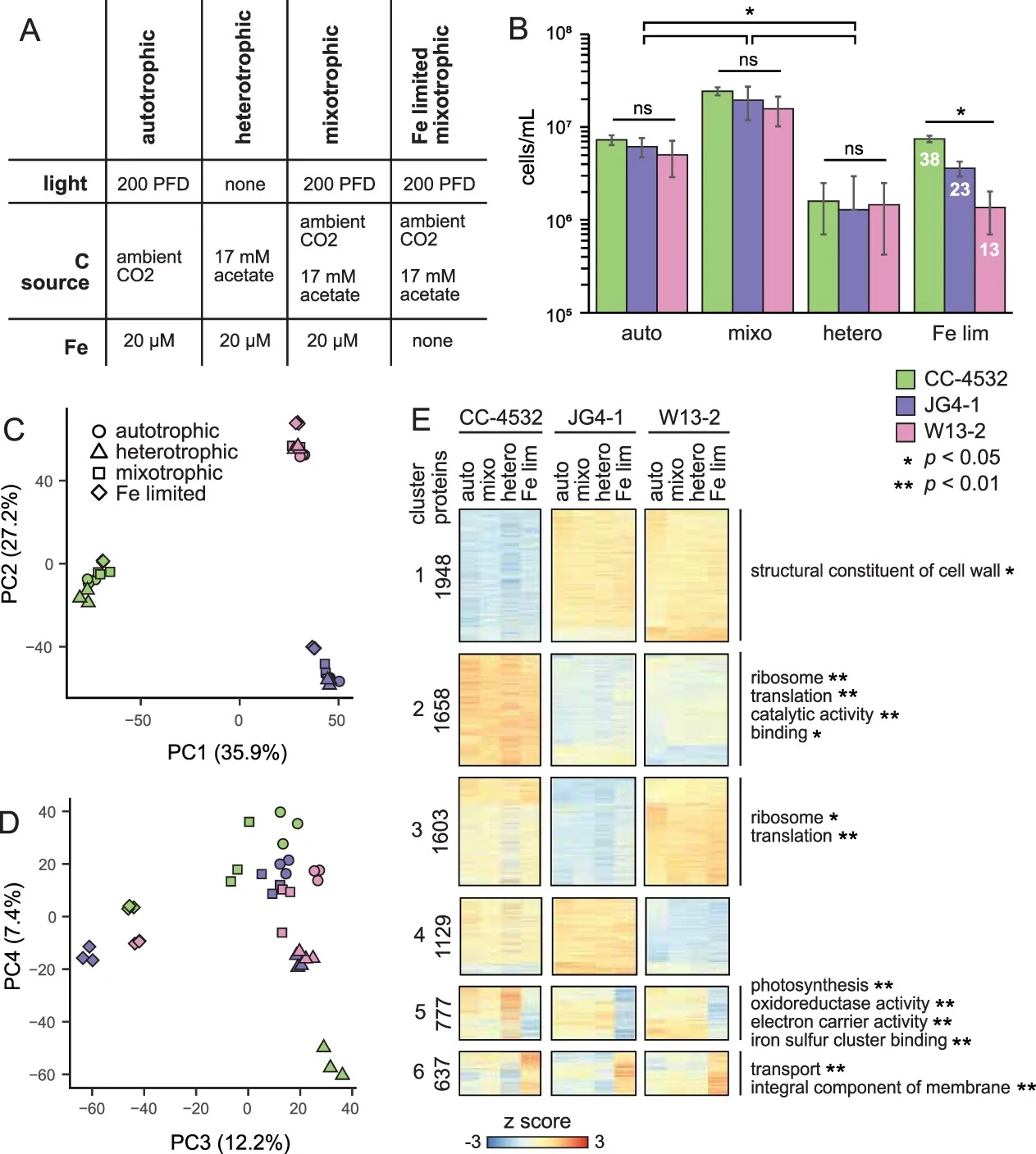
Comparison of *C. reinhardtii* strains CC-4532 (laboratory reference), JG4-1 (field), and W13-2 (field) under different trophic conditions. (A) Trophic conditions examined. (B) Growth of each strain under each condition, as collected for proteomic analyses. For the iron limited condition, numbers in bars indicate the percent of growth relative to an iron replete control inoculated in parallel. (C, D) PCA of protein abundances illustrating variance in proteomes of each strain under each condition. PC1 and PC2 highlight differences between strains (C), while PC3 and PC4 capture differences between conditions (D). (E) K-means clustered heatmap of normalized protein abundances (z-scores of mean abundance values) illustrating distinct patterns across these proteomes, annotated with significantly enriched GO terms indicating the prevalence of proteins with related functions in a cluster.

Metabolic activities drive ecological relationships. *C. reinhardtii* grows autotrophically in the presence of light and absence of acetate, heterotrophically in the presence of acetate and absence of light, and mixotrophically in the presence of both light and acetate. To examine differences between field strains and their laboratory-adapted counterparts across their metabolic landscape, we grew axenic cultures of two distinct GTA strains (JG4-1 and W13-2) and a reference laboratory strain (CC-4532) under four different conditions (nutrient-replete autotrophic, heterotrophic, mixotrophic, and iron-limited mixotrophic growth, Fig. 2A and B). For all strains, we observed the highest growth under mixotrophy, moderate growth under autotrophy, and low growth under heterotrophy. The only significant differences between strains were under iron limitation, where we observed growth defects of 62% from CC-4532, 78% from JG4-1, and 87% from W13-2.

### Variance across proteomes is dominated by differences between strains

To capture a comprehensive window into their biology, we examined the proteomes of each *C. reinhardtii* strain grown under each trophic condition. We detected similar ranges of protein abundances from each sample, indicating that we recovered comparable proteomes across this dataset (Fig. S7). Principal component analysis (PCA) illustrated that the variance among these proteomes was dominated by differences between strains that persisted across all conditions (Fig. 2C) but also captured differences between conditions (Fig. 2D). PC1 separated CC-4532 from both field strains, while PC2 separated all three strains. PC3 separated iron limitation from the other conditions, showing that across all strains, growth under iron limitation changed the proteome more than any of the other trophies. PC4 separated light (autotrophic and mixotrophic) from dark (heterotrophic) growth; notably, this separation was far more pronounced for CC-4532 than either of the field strains, indicating that heterotrophic growth was accompanied by more proteomic changes for this laboratory strain than field strains. Additional minor principal components did not reveal further patterns.

K-means clustering revealed six protein clusters with different abundance patterns (Fig. 2E), echoing the differences captured by the PCA. Like PC1, the largest clusters (clusters 1 and 2) distinguished CC-4532 from both field strains, across all trophic conditions. Like PC2, clusters 3 and 4 distinguished individual field strains as end members. Like PC3 and 4, clusters 5 and 6 captured differences between conditions, with iron limitation standing out across all strains, and heterotrophy standing out for just CC-4532. To examine the functional content, we identified gene ontology (GO) terms significantly enriched in each protein cluster. In cluster 1, which contains proteins exhibiting higher abundances in both field strains than CC-4532, the only enriched GO term was “structural constituent of the cell wall.” In cluster 2, which contains proteins exhibiting lower abundances in both field strains than CC-4532, and cluster 3, which contains proteins lower in JG4-1 than the other two strains, GO terms relating to ribosomes and translation were enriched. In cluster 5, which contains proteins exhibiting lower abundances under iron limitation, and cluster 6, which contains proteins exhibiting higher abundances under iron limitation, enriched GO terms reflected known responses to iron limitation such as decreased photosynthesis and increased transporters.

The proteomic differences between strains cannot be explained by differences in gene copy number. Genome sequence reads from JG4-1 and W13-2 mapped onto the CC-4532 reference genome largely fell in a normal distribution, and gene coverage did not broadly correlate with protein abundance (Fig. S8A and B). However, a small subset (three proteins in JG4-1 and seven proteins in W13-2) exhibited significantly higher gene coverage and protein abundance (Fig. S8C). Only one of these proteins (PGK2) is annotated with a gene symbol in the *C. reinhardtii* v6.1 genome. Large-scale genome duplications that can span tens to hundreds of kilobases have been identified in both laboratory and field strains of *C. reinhardtii* [4], although the effect of gene copy number variants on protein abundance is not known.

### Heterogeneity in carbon and energy metabolism

**Figure 3:**
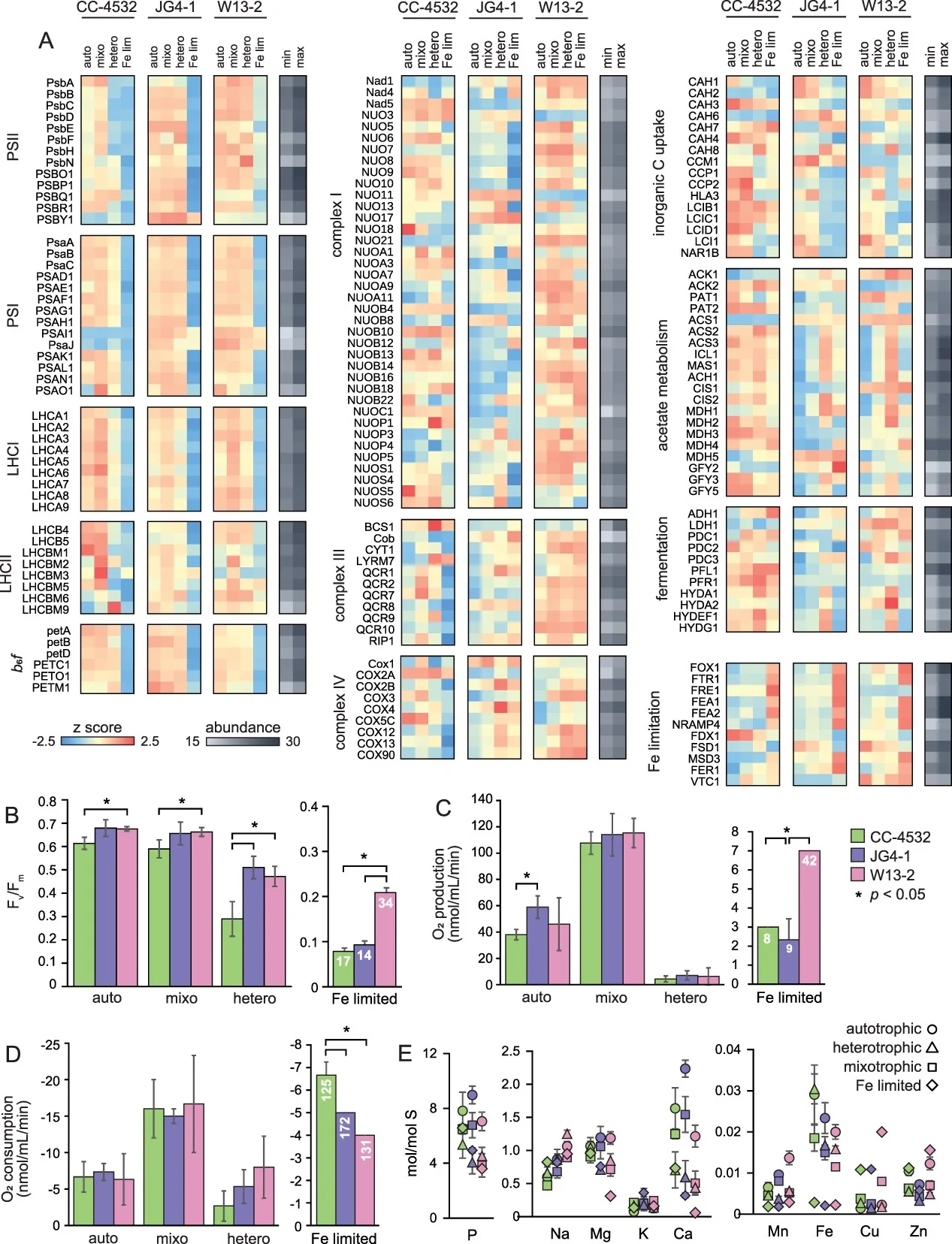
Metabolism and nutrition of *C. reinhardtii* strains. (A) Normalized abundance (z-scores of mean abundance values) and abundance range (minimum and maximum abundance values) of proteins involved in metabolism and nutrition. (B, C) Photosynthetic activity measured by variable Chl fluorescence (B) and O_2_ production rate (C). (D) Respiratory activity measured by O_2_ consumption rate. For the Fe limited condition, numbers in bars indicate the percent of activity per cell relative to an iron replete control inoculated in parallel. (E) Elemental content of biomass measured by ICPMS, presented as a ratio to sulfur as a proxy for normalizing to total biomass.

To explore how the biology underpinning different metabolic modes varied across strains, we examined the abundances of known proteins directly involved in photosynthesis, respiration, carbon metabolism, and iron homeostasis (Fig. 3A). To understand how these differences in protein abundances might relate to metabolic activities, we measured photosynthetic capacity (Fig. 3B and C), respiratory capacity (Fig. 3D), and biomass elemental content (Fig. 3E) of each strain grown under each condition.

The field strains expressed higher abundances of photosynthetic reaction center proteins, particularly PSBY1, PSAI1, and PsaJ. While CC-4532 decreased photosynthesis protein abundances in the dark, the field strains maintained that machinery at comparable levels as in the light. All strains had less photosynthesis proteins under iron limitation, although that decrease was less dramatic for W13-2 than for the other two strains. These differences in photosynthesis proteins correlated with differences in photosynthetic activity measurements—the field strains exhibited higher F_v_/F_m_ and O_2_ production rates than did CC-4532, particularly when grown heterotrophically, and W13-2 maintained higher photosynthetic activity under iron limitation than did the other two strains.

Unlike the photosynthesis proteins, abundances of mitochondrial electron transport chain proteins were not reflected in higher respiratory activity. W13-2 had higher abundances of respiratory complex I, III, and IV subunits across all conditions, but did not exhibit higher respiratory activity except under heterotrophic growth. Under iron limitation, all strains decreased several complex I subunits, while only CC-4532 decreased complex III and IV subunits. Nonetheless, all strains had higher per cell respiration rates under iron limitation than did their corresponding iron replete control.

Although all strains were grown under the same atmospheric CO_2_ level (0.04%), we observed differences in the abundance of proteins involved in inorganic carbon uptake. CC-4532 expressed higher abundances of proteins reflecting low CO_2_-induced carbon concentrating mechanisms (CCMs), including CCP2, HLA3, LCIB1, LCIC1, and LCID1. Different carbonic anhydrases exhibited distinct abundance patterns between strains and conditions. For example, among the chloroplast-localized carbonic anhydrases CAH3, CAH6, and CAH7, CAH3 was most abundant in CC-4532, while CAH6 was more abundant in the field strains, and CAH7 increased under the lower growth conditions (heterotrophy and iron limitation) for all strains.

Many proteins involved in acetate metabolism and fermentation pathways were more abundant in CC-4532 than in the field strains and were highly expressed across all conditions for CC-4532 but only under heterotrophic growth for the field strains. The higher abundance of acetate metabolism proteins observed in CC-4532 was not reflected in higher respiration rates. However, all strains exhibited higher per cell respiration rates under heterotrophy, the condition with the highest abundance of acetate metabolism proteins in the field strains. We again observed distinct abundance patterns from different isoforms of proteins including the acetate kinases, acetyl-CoA synthetases, citrate synthases, malate dehydrogenases, and acetate transporters.

Although differences between trophic conditions were relatively minor in the scope of our full proteomes, they reflect dramatically different lifestyles. With the combination of proteomic and activity data, this dataset provides a rich window into strain-level metabolic variation in *C. reinhardtii*, and this variation suggests field strains are more reliant on and more adept at photosynthesis under atmospheric CO_2_, while CC-4532 is more adapted to life in culture with acetate. The field strains maintained photosynthesis proteins under heterotrophic growth but did not maintain acetate metabolism proteins under autotrophic growth; CC-4532 exhibited the opposite and may rely on a greater abundance of CCM proteins.

### W13-2 exhibits unique response to iron limitation

In the proteomes of all strains under iron limitation, we observed known hallmarks of iron deficiency including dramatic increases of proteins involved in iron uptake FOX1, FTR1, FEA1, FEA2, and NRAMP4; decrease of ferredoxin FDX1; decrease of the iron superoxide dismutase FSD1 and its replacement with the manganese superoxide dismutase MSD3; and increase of ferritin FER1 (Fig. 3A) [39]. Notably, the ferric reductase FRE1, a sentinel gene upregulated upon iron deficiency, increased in CC-4532 and JG4-1, but not in W13-2. Taken together with the growth (Fig. 2B) and activity (Fig. 3B and C) data, this suggests a distinct response to iron limitation for W13-2.

Biomass iron content showed a comparable scarcity in all strains under iron limitation, suggesting no major differences in iron uptake or availability (Fig. 3E). Esteves *et al.* [40] previously reported on the elemental composition of biomass of other *C. reinhardtii* field strains grown under autotrophic, mixotrophic, and nutrient-limited conditions. Consistent with their findings, we observed higher accumulation of most elements except copper in biomass grown autotrophically than mixotrophically and higher accumulation of copper under iron limitation. However, while they observed dramatic (>10-fold) differences in elemental composition between strains, we did not observe differences greater than 3-fold for any elements, with the exception of calcium in W13-2 under iron limitation.

Iron homeostasis in *C. reinhardtii* centers on iron storage and mobilization from lysosome-related organelles resembling acidocalcisomes, with high levels of calcium and polyphosphate [39, 41]. Under iron limitation, we observed decreased calcium content of biomass for all strains, with vanishingly low calcium remaining in W13-2. This could be a signal of acidocalcisomes being emptied to mobilize stored iron. Interestingly, the vacuolar transporter chaperone VTC1, responsible for polyphosphate accumulation within acidocalcisomes [41], was more abundant in W13-2 than in the other two strains, across all conditions. Most other candidate acidocalcisome proteins identified by Long *et al.* [42] did not exhibit the same pattern, though many were more abundant under iron limitation in all strains (Fig. S9).

Future work is required to reach a mechanistic understanding of the unique response to iron limitation exhibited by W13-2. However, the phenomenon we captured—that W13-2 grew the least but maintained the most photosynthetic activity under iron limitation—illustrated a tradeoff between growth and maintenance, highlighting an important dimension of phenotype plasticity in responses to nutrient deficiency.

### Field strains have more abundant cell wall proteins and higher cell wall permeability

To explore major differences between strains expressed across all trophic conditions, we followed the leads suggested by our GO analysis (Fig. 2E). The cluster of proteins more abundant in both field strains than CC-4532 was enriched with cell wall proteins; we therefore examined the abundance patterns of various proteins associated with the cell wall, including matrix metalloproteinases, pherophorins, and other hydroxyproline-rich glycoproteins. To complement these proteomic observations with an empirical measure of cell wall function, we assayed its capacity to protect the cell membrane from detergent solubilization.

**Figure 4:**
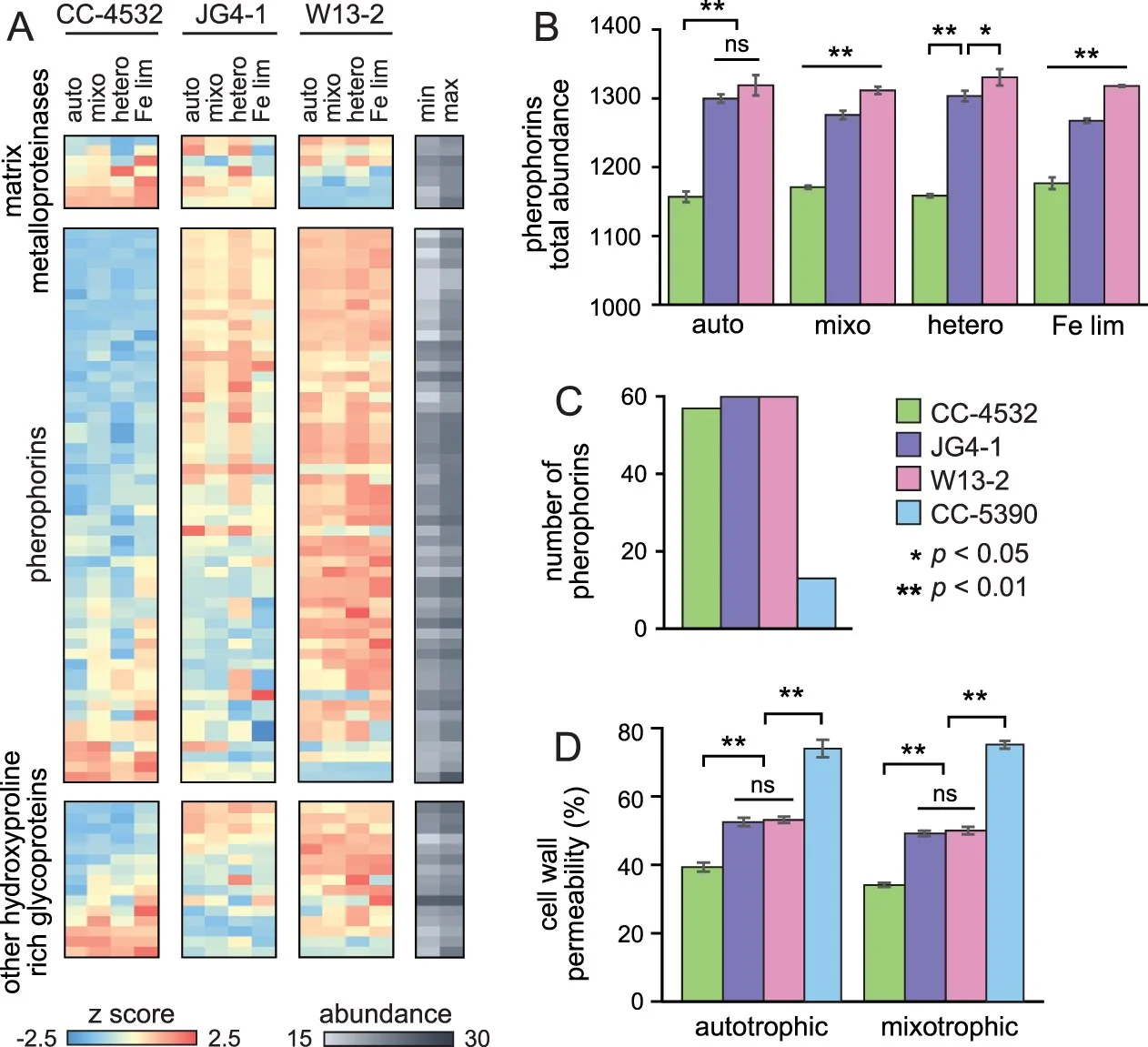
Cell wall proteins and permeability of *C. reinhardtii* strains. (A) Normalized abundance (z-scores of mean abundance values) and abundance range (minimum and maximum abundance values) of cell wall proteins detected in all strains and conditions. (B) Total abundance (sum of abundance values) of pherophorins in each proteome. (C) Count of pherophorins detected in each strain across all conditions. CC-5390, a cell wall-deficient laboratory strain, is included for comparison. (D) Cell wall permeability measured by membrane susceptibility to solubilization by detergent, presented as the percent of absorbance at 680 nm released from cells upon Triton X-100 treatment.

Each strain exhibited distinct patterns of cell wall proteins, with notably higher abundances in both field strains than in CC-4532, especially of pherophorins (Fig. 4A and B). The high total pherophorin content in the field strains was not due to dominance of individual extremely abundant proteins; in fact, the highest abundance of any one pherophorin was from CC-4532. Rather, the field strains achieved higher total pherophorin content through higher abundance of a greater diversity of pherophorins. While only 5 pherophorins were consistently more abundant in CC-4532, as many as 54 were consistently more abundant in one or both field strains. Only a small subset of these proteins was detected in a prior dataset generated with CC-5390, a cell wall-deficient laboratory strain [43] (Fig. 4C). While CC-5390 exhibited the highest cell wall permeability, both field strains exhibited significantly higher cell wall permeability than did CC-4532 (Fig. 4D).

The observation of highly abundant pherophorins in field strains invokes known transitions toward multicellularity. *C. reinhardtii* is a member of the Volvocine lineage, a set of related algae spanning unicellular species (e.g. *C. reinhardtii*), undifferentiated multicellular colonial species (e.g. *Gonium pectorale*), and multicellular species with differentiated cell types (e.g*. Volvox carteri*). Studies on the evolution of multicellularity in this lineage identified 12 major developmental transitions, 2 of which center on pherophorins [6, 7]. Between *C. reinhardtii* and *G. pectorale*, existing pherophorins are co-opted into new roles without genomic expansion; between *G. pectorale* and *V. carteri*, pherophorin genes are greatly expanded but retain close homology to their unicellular relatives [44, 45]. Across these transitions, pherophorin adaptive plasticity facilitates remodeling the unicellular cell wall into an increasingly extensive and developmentally complex extracellular matrix.

Pherophorins are also involved in multicellular communal behaviors of *C. reinhardtii* itself. Triggered by a wide range of biotic and abiotic stressors, *C. reinhardtii* will form multicellular structures that have a survival advantage over single cells [46]. Under selection pressure, this behavior develops into a heritable multicellular lifecycle [8, 9]. Different pherophorins were identified with both pro- and anti-aggregative roles in this behavior, leading to the hypothesis that their equilibrium is regulated to control the formation of multicellular structures [47]. We saw both roles represented among the pherophorins more abundant in the field strains, consistent with this hypothesis. Their high expression of diverse pherophorins under baseline conditions—not as a stress response—suggests the field strains are adapted to operate in multicellular units.

Cell wall permeability relates directly to biotic interactions. As primary producers, algae provide growth substrates to other organisms. Algal symbionts of lichens and invertebrates release much more fixed carbon than do their free-living relatives [48]. While the permeability of CC-5390 is the outcome of engineered cell wall deficiency reflected in a dearth of cell wall proteins, the permeability of the field strains is a natural adaptation accompanied by a complex array of abundant cell wall proteins. Understanding that these field strains coexisted with specific symbionts in the field, we interpreted their high permeability as a hallmark of life in symbiosis.

### Field strains have less abundant ribosomal proteins but more robust autotrophic growth

**Figure 5:**
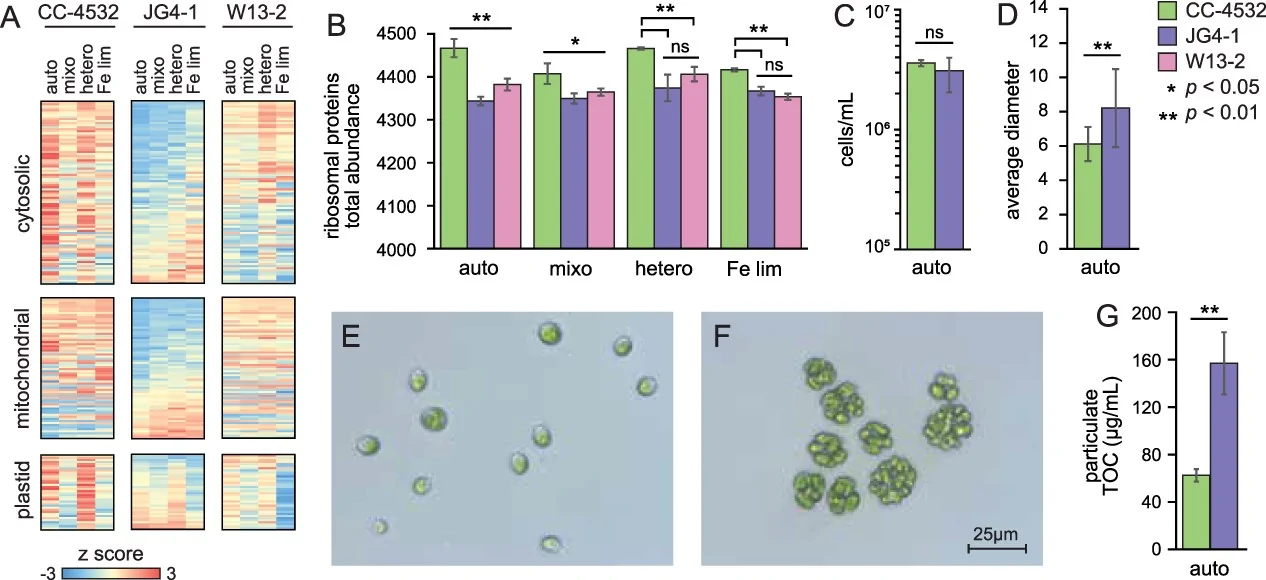
Ribosomal proteins and growth of *C. reinhardtii* strains. (A) Normalized abundance (z-scores of mean abundance values) of ribosomal proteins detected in all strains and conditions. (B) Total abundance (sum of abundance values) of ribosomal proteins detected in each strain across all conditions. (C) Growth of autotrophic CC-4532 and JG4-1 cultures measured by cell counts. (D) Average size of autotrophic CC-4532 and JG4-1 cells. (E, F) Light microscopy of autotrophic CC-4532 (E) and JG4-1 (F) cultures. (G) Biomass TOC of autotrophic CC-4532 and JG4-1 cultures. Despite a lower abundance of ribosomal proteins, JG4-1 exhibits more robust autotrophic growth when measured as biomass TOC rather than cell counts.

The clusters of proteins less abundant in both field strains than in CC-4532 and less abundant in JG4-1 than in the other two strains were enriched in GO terms relating to ribosomes and translation (Fig. 2E). We therefore examined the abundance patterns of ribosomal proteins. Under all trophic conditions, we observed significantly lower ribosomal protein abundance in both field strains than in CC-4532 (Fig. 5A and B).

Ribosome content is generally understood to be positively correlated with microbial growth rate [49, 50]. We did not observe significant differences in growth as measured by cell counts (Fig. 2B). However, we noted that the field strain cells are, on average, bigger (Fig. 5D) and clumpier (Fig. 5E and F) than CC-4532; thus, cell counts tend to underrepresent their growth in terms of biomass generated.

To compare growth more directly, we focused on autotrophic JG4-1 and CC-4523 since they exhibited the largest disparity in ribosomal protein abundance. Once again, our cell counts were not significantly different (Fig. 5C). However, TOC measurements demonstrated that JG4-1 cultures generated more than double the biomass of CC-4532 (Fig. 5G). Taken together with the observation that CC-4532 expresses more proteins reflecting CO_2_ limitation (Fig. 3A), this suggests that JG4-1 is more adept at autotrophic growth under atmospheric CO_2_ levels than the laboratory strain is. The apparent departure from the microbial growth law with respect to ribosomal proteins could be due to heterogeneity of growth rates within clumps, where cells on the periphery are more metabolically active than are cells in the center [51].

### JG4-1 clumping phenotype arises from multiplet division not just aggregation

**Figure 6:**
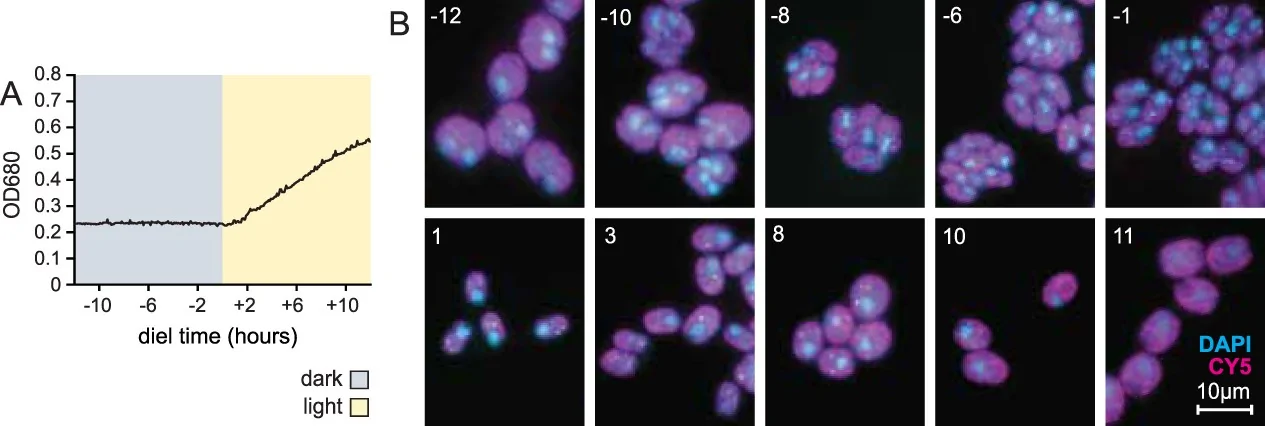
Synchronized cell cycle of JG4-1. (A) Optical density of photobioreactor cultures demonstrating growth in the light and quiescence in the dark. (B) Fluorescence microscopy around the cell cycle demonstrating multiplet division in the night and dispersal upon daybreak. DAPI staining is shown in cyan; Chl fluorescence is shown in magenta. Numbers indicate timepoint in diel time (hours relative to lights on).

The clumpy growth phenotype observed in the field strains (Fig. 5F) complements their proteomic signatures of multicellular behavior (Fig. 4A–C). To better understand the ontogeny of this phenotype, we examined the cell cycle of JG4-1. We generated a synchronized population by growing them autotrophically in a photobioreactor under diurnal light and compared them to the previously characterized cell cycle of laboratory strain CC-5390 [32, 34]. Like CC-5390, JG4-1 cells grew autotrophically during the light phase (Fig. 6A) and divided upon nightfall. However, under the same conditions that yielded exactly two daughter cells for CC-5390, JG4-1 underwent multiplet division, yielding clusters of daughter cells (Fig. 6B). While CC-5390 daughter cells separated promptly after division, the JG4-1 clusters remained intact for the duration of the dark phase, separating only upon daybreak and even then separating incompletely. These clusters of cells observed in the context of the cell cycle resembled the clumps observed in unsynchronized JG4-1 cultures (Fig. 5F), suggesting a similar origin.

Multicellular structures arising from cell division without separation (palmelloids) and those arising from separate individuals coming together (aggregates) have different implications. Both can provide communal survival advantage over individual cells [47], and both are known routes to the establishment of multicellularity [52, 53]. However, they are subject to different evolutionary dynamics. Multicellular groups with a unicellular origin experience stronger selection between groups than within groups, favoring the development of more cooperativity. In contrast, the initial diversity within an aggregated group enhances internal competition and selection, and thus the potential development of cheater lines. In laboratory strains of *C. reinhardtii*, the palmelloid phenotype is induced as a stress response [54–56] and can be selected for as a heritable trait [8, 9]. Like the high expression of pherophorin proteins, its prevalence in the field strains under baseline conditions may reflect adaptation to multicellular cooperativity.

### Natural *C. reinhardtii* symbiont *Brevundimonas sp.* Bl-2 is supported by live algal partners

**Figure 7:**
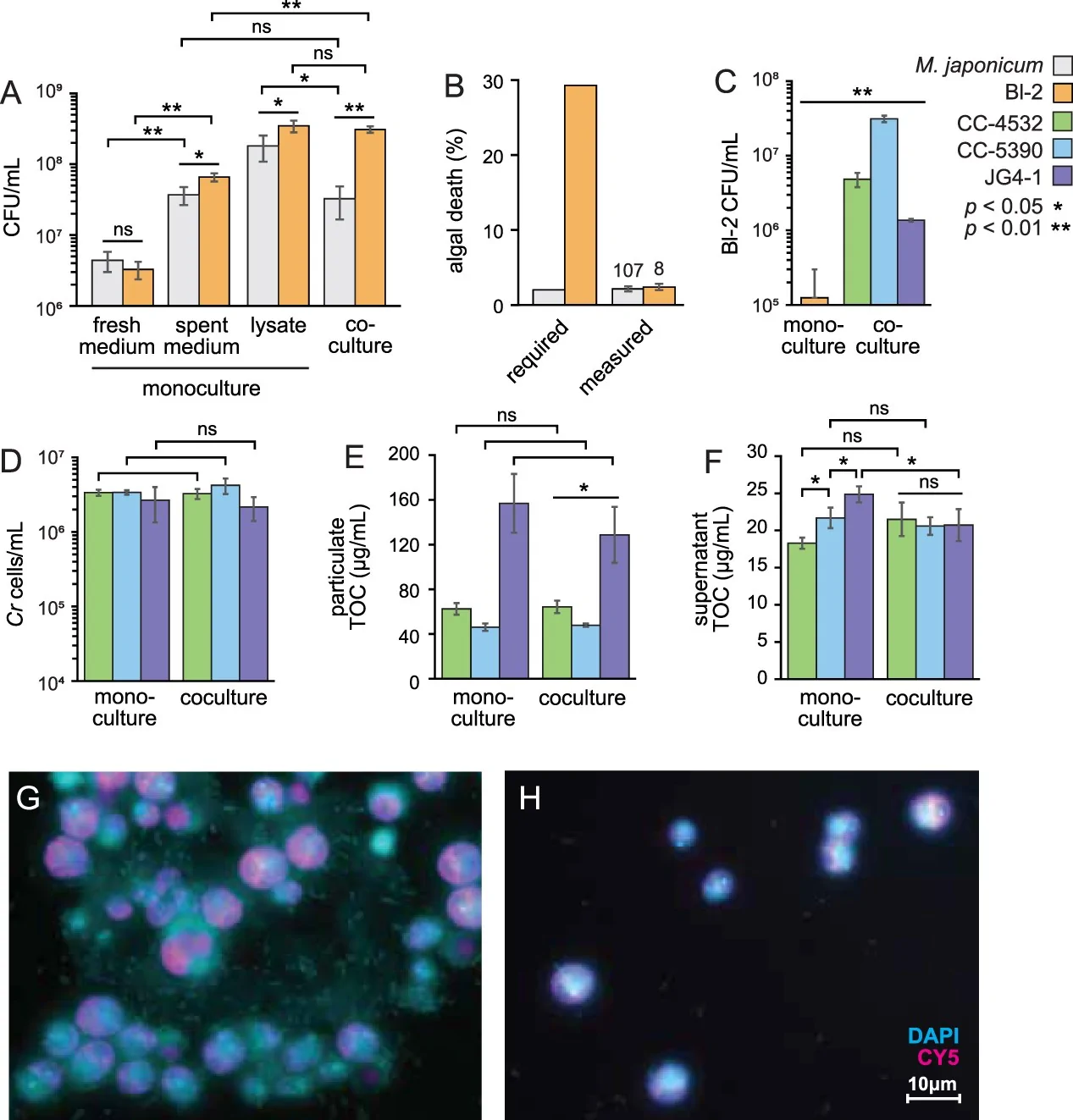
*Chlamydomonas–Brevundimonas* symbiosis. (A) Growth of Bl-2 when inoculated into minimal medium with no organic carbon source, in spent medium from *C. reinhardtii*, in coculture with *C. reinhardtii*, and in *C. reinhardtii* cell lysate, plotted with previously published data of model bacterial partner *M. japonicum* [11]. This experiment was conducted with autotrophic *C. reinhardtii* lab strain AHS-32 and bacteria inoculated at much higher density than the cocultures shown in panels (C–F) to enable direct comparison to the *M. japonicum* system. (B) Algal death required to account for observed growth of each bacterium in coculture if all growth was derived from algal necromass, calculated based on bacterial growth on algal lysate. Actual algal death measured by CellTox fluorescence. Numbers above bars indicate the percent of required values realized by measured values. (C) Growth of Bl-2 in monoculture vs. coculture with different autotrophic *C. reinhardtii* strains. (D, E) Growth of autotrophic *C. reinhardtii* strains in monoculture vs. coculture, measured by cell counts (D) and biomass TOC (E). (F) TOC of spent medium from autotrophic *C. reinhardtii* strains in monoculture vs. coculture demonstrating strain-level differences in exudation. (G, H) Fluorescence microscopy of Bl-2 cocultures with JG4-1 (G) and CC-5390 (H). DAPI staining is shown in cyan; Chl fluorescence is shown in magenta.

To examine a symbiosis between *C reinhardtii* and their bacterial associates, we chose isolate *Brevundimonas sp.* Bl-2, which coenriched from the field with JG4-1. We began by comparing this symbiosis to previous work presented by Dupuis *et al.* [11] on the model algal–bacterial symbiosis between *C. reinhardtii* and *Mesorhizobium japonicum* (Fig. 7A and B). Both *Brevundimonas* and *Mesorhizobium* are aerobic heterotrophic Alphaproteobacteria [57]; while *Brevundimonas* is repeatedly implicated in ecological association with *C. reinhardtii* (Fig. 1A, [37, 38]), *Mesorhizobium* is not. With *M. japonicum*, we observed similar growth yields in *C. reinhardtii* spent medium as in coculture with live *C. reinhardtii*, and significantly higher growth on *C. reinhardtii* lysate. Based on the bacterial growth yields on lysate, we calculated the amount of algal lysis required to explain the bacterial growth in coculture and found that it was equal to the algal death occurring in those cultures. Therefore, we concluded that *M. japonicum* growth in coculture was predominantly due to substrates derived from algal death rather than transferred from live partners. In contrast, Bl-2 exhibited significantly more growth in coculture than in spent medium, demonstrating a much more active symbiosis. Moreover, only 8% of the Bl-2 growth in coculture could be attributed to algal lysis, demonstrating that Bl-2 growth must be supported by live algal partners.

To investigate how differences in algal cell wall affect organic carbon exudation, we measured the TOC of spent medium from different *C. reinhardtii* strains in monoculture and coculture with Bl-2 (Fig. 7F). Among the laboratory strains, we observed significantly higher TOC released from CC-5390 than from CC-4532, as expected based on their relative cell wall permeability (Fig. 4D). However, TOC released from JG4-1 was higher still, demonstrating that exudation of organic carbon was not simply a function of permeability. Notably, JG4-1 was the only strain with a significant decrease in spent medium TOC between monoculture and coculture, likely reflecting bacterial consumption of exuded substrates.

Species- and even strain-level variations are known to impact the outcomes of host–microbe interactions [58]. Our data show that interactions in the *C. reinhardtii* phycosphere are no exception. Unlike *M. japonicum*, Bl-2 grows on substrates provided by live algal partners. Unlike the laboratory strains of *C. reinhardtii*, JG4-1 exudes substrates consumed by Bl-2 in higher fluxes. Thus, the trophic transfers occurring between these different pairings of organisms are distinct. As the JG4-1 and Bl-2 pairing originated in the environment, their interaction may reflect adaptions to living in partnership with each other.

### Cocultures of JG4-1 and Bl-2 form shared biofilms

Coculture with Bl-2 did not impact the growth of any *C. reinhardtii* strain (Fig. 7D and E), and all *C. reinhardtii* strains supported robust Bl-2 growth (Fig. 7C). Among the laboratory strains, the magnitude of Bl-2 growth correlated with cell wall permeability (Fig. 4D) and organic carbon exudation (Fig. 7F), providing an additional line of evidence that this symbiosis is supported by algal exudates. Although we observed less Bl-2 growth with its native partner JG4-1 than with either of the laboratory strains (Fig. 7C), we note that bacterial growth was measured by counting colony-forming units (CFUs) from serially diluted cultures spotted onto agar plates, another growth metric confounded by the clumpiness of JG4-1. Microscopy revealed that cocultured JG4-1 and Bl-2 formed shared biofilms with abundant bacteria embedded in extracellular polymeric substances (Fig. 7G), whereas cocultures with laboratory strains (Fig. 7H) and monocultures did not.

Biofilms are dense consortia held together by an extracellular matrix and have long been recognized for emergent properties that distinguish them from communities of free-living planktonic cells [59]. The matrix environment provides stability, protection, and structure, within which heterogeneous nutrient dynamics, physiologies, and interactions operate. Up to 80% of microbial life on Earth, and essentially all microbial life in soil, is estimated to exist in biofilms [60]. Recognizing that cocultures of Bl-2 with its native algal partner JG4-1 develop biofilms while cocultures with laboratory strains do not highlights the importance of studying natural pairings to capture interactions in their ecologically relevant form. While the experiments presented here were all conducted in liquid culture, other cultivation conditions even more conducive to biofilm formation (solid substrates, soil analogs) may be more representative of their natural form.

## Conclusions

We examined field strains of *C. reinhardtii* for insights into their ecology and found several lines of evidence demonstrating their participation in multicellular units. They expressed high levels of diverse pherophorin proteins similar to major developmental transitions in the evolution of multicellularity. Their palmelloid clumping phenotype resembled a multicellular lifecycle. Their permeable cell walls and association with bacteria supported by their exudates highlighted their adaptation to symbioses with other organisms, and their formation of a shared biofilm matrix with those potential symbionts showed that their interactions involve collective organization. Whether understood as individuals or communities, multicellular units like palmelloids and biofilms have emergent properties beyond the sum of their unicellular constituents. Thus, we must consider their biology through the lens of their interactions, rather than as unicellular individuals, to understand how it underpins their ecology.

Even on the cell biological level, the characteristics that distinguished laboratory and field strains underscored the importance of interactions, since the most prominent difference we observed concerned their cell walls. As the exterior of a cell, the cell wall controls its recognizability to other organisms. As the barrier between a cell and the world around it, cell walls distinguish a unicellular individual from its environment and mediate the crosstalk between those entities including the release of exudates. In the transition from unicellularity to multicellularity, including the multicellularity of palmelloids and biofilms, the cell wall is remodeled into an extracellular matrix. Thus, strain-level differences in cell wall likely shape and coevolve with interactions.

To apply the *C. reinhardtii* model system to ecological questions—in particular, interactions between *C. reinhardtii* and other organisms—is a growing area of research interest [36]. As such, we emphasize the importance of considering multicellular dynamics and strain-level differences in adaptation to symbiosis when designing such research. The observations presented here are limited to the small number of strains examined; therefore, we cannot distinguish unique characteristics of these strains from universal characteristics of field vs. laboratory strains. Nonetheless, the differences we observed underscore the importance of choosing the right strain for the task, depending on the questions and methods involved. Some frameworks developed with laboratory strains are readily extended to field strains, demonstrated here by genomic and proteomic data mapped to the CC-4532 reference genome. Others are not, demonstrated here by growth measures (algal cell counts and bacterial CFUs) that work well with laboratory strains but are confounded with field strains. Well-characterized laboratory strains amenable to growth in homogenous culture provide high resolving power for dissecting biological processes at the mechanistic level; field strains provide a window into the ecological relevance lost over decades of adaptation to life in the laboratory.

## Supplementary Material

Supplementary_material_wrag202

## Data Availability

Proteomic data is deposited in the MassIVE repository under accession number MSV000100518. Amplicon sequencing data is deposited on eDNAexplorer (https://www.ednaexplorer.org/profile/clwgz33az0000x0cwfurz99ur). All other data is available in Supplementary Tables S1–S15.

## References

[1] Salomé PA, Merchant SS. A series of fortunate events: introducing *Chlamydomonas* as a reference organism. *Plant Cell* 2019;31:1682–707. 10.1105/tpc.18.00952 31189738 PMC6713297

[2] Sasso S, Stibor H, Mittag M, et al. From molecular manipulation of domesticated *Chlamydomonas reinhardtii* to survival in nature. *eLife* 2018;7:e39233. 10.7554/eLife.39233 30382941 PMC6211829

[3] Mocali S, Benedetti A. Exploring research frontiers in microbiology: the challenge of metagenomics in soil microbiology. *Res Microbiol* 2010;161:497–505. 10.1016/j.resmic.2010.04.010 20452420

[4] Flowers JM, Hazzouri KM, Pham GM, et al. Whole-genome resequencing reveals extensive natural variation in the model green alga *Chlamydomonas reinhardtii*. *Plant Cell* 2015;27:2353–69. 10.1105/tpc.15.00492 26392080 PMC4815094

[5] Craig RJ, Böndel KB, Arakawa K, et al. Patterns of population structure and complex haplotype sharing among field isolates of the green alga *Chlamydomonas reinhardtii*. *Mol Ecol* 2019;28:3977–93. 10.1111/mec.15193 31338894

[6] Jiménez-Marín B, Olson BJSC. The curious case of multicellularity in the Volvocine algae. *Front Genet* 2022;13:787665. 10.3389/fgene.2022.787665 35295942 PMC8919427

[7] Kirk DL . A twelve-step program for evolving multicellularity and a division of labor. *BioEssays* 2005;27:299–310. 10.1002/bies.20197 15714559

[8] Chen ICK, Khatri S, Herron MD, et al. Genetic predisposition toward multicellularity in *Chlamydomonas reinhardtii*. *Genome Biol Evol* 2025;17:evaf090. 10.1093/gbe/evaf090 40380887 PMC12124119

[9] Ratcliff WC, Herron MD, Howell K, et al. Experimental evolution of an alternating uni- and multicellular life cycle in *Chlamydomonas reinhardtii*. *Nat Commun* 2013;4:2742. 10.1038/ncomms3742 24193369 PMC3831279

[10] Gallaher SD, Fitz-Gibbon ST, Glaesener AG, et al. *Chlamydomonas* genome resource for laboratory strains reveals a mosaic of sequence variation, identifies true strain histories, and enables strain-specific studies. *Plant Cell* 2015;27:2335–52. 10.1105/tpc.15.00508 26307380 PMC4815092

[11] Dupuis S, Lingappa UF, Mayali X, et al. Scarcity of fixed carbon transfer in a model microbial phototroph-heterotroph interaction. *ISME J* 2024;18:wrae140. 10.1093/ismejo/wrae140 39046282 PMC11316394

[12] Ford SA, Craig RJ, Ness RW. A novel method for identifying *Chlamydomonas reinhardtii* (Chlorophyta) and closely related species from nature. *J Phycol* 2023;59:281–8. 10.1111/jpy.13306 36453860

[13] Kan Y, Pan J. A one-shot solution to bacterial and fungal contamination in the green alga *Chlamydomonas reinhardtii* culture by using an antibiotic cocktail. *J Phycol* 2010;46:1356–8. 10.1111/j.1529-8817.2010.00904.x

[14] Arkin AP, Cottingham RW, Henry CS, et al. KBase: the United States Department of Energy Systems Biology Knowledgebase. *Nat Biotechnol* 2018;36:566–9. 10.1038/nbt.4163 29979655 PMC6870991

[15] Menzel P, Ng KL, Krogh A. Fast and sensitive taxonomic classification for metagenomics with kaiju. *Nat Commun* 2016;7:11257. 10.1038/ncomms11257 27071849 PMC4833860

[16] Li D, Liu CM, Luo R, et al. MEGAHIT: an ultra-fast single-node solution for large and complex metagenomics assembly via succinct de Bruijn graph. *Bioinformatics* 2015;31:1674–6. 10.1093/bioinformatics/btv033 25609793

[17] Gurevich A, Saveliev V, Vyahhi N, et al. QUAST: quality assessment tool for genome assemblies. *Bioinformatics* 2013;29:1072–5. 10.1093/bioinformatics/btt086 23422339 PMC3624806

[18] Alneberg J, Bjarnason BS, De Bruijn I, et al. Binning metagenomic contigs by coverage and composition. *Nat Methods* 2014;11:1144–6. 10.1038/nmeth.3103 25218180

[19] Kang DD, Froula J, Egan R, et al. MetaBAT, an efficient tool for accurately reconstructing single genomes from complex microbial communities. *PeerJ* 2015;3:e1165. 10.7717/peerj.1165 26336640 PMC4556158

[20] Sieber CMK, Probst AJ, Sharrar A, et al. Recovery of genomes from metagenomes via a dereplication, aggregation and scoring strategy. *Nat Microbiol* 2018;3:836–43. 10.1038/s41564-018-0171-1 29807988 PMC6786971

[21] Parks DH, Imelfort M, Skennerton CT, et al. CheckM: assessing the quality of microbial genomes recovered from isolates, single cells, and metagenomes. *Genome Res* 2015;25:1043–55. 10.1101/gr.186072.114 25977477 PMC4484387

[22] Chaumeil PA, Mussig AJ, Hugenholtz P, et al. GTDB-Tk v2: memory friendly classification with the genome taxonomy database. *Bioinformatics* 2022;38:5315–6. 10.1093/bioinformatics/btac672 36218463 PMC9710552

[23] Brettin T, Davis JJ, Disz T, et al. RASTtk: a modular and extensible implementation of the RAST algorithm for building custom annotation pipelines and annotating batches of genomes. *Sci Rep* 2015;5:art. 1. 10.1038/srep08365 PMC4322359 25666585

[24] Shaffer M, Borton MA, McGivern BB, et al. DRAM for distilling microbial metabolism to automate the curation of microbiome function. *Nucleic Acids Res* 2020;48:8883–900. 10.1093/nar/gkaa621 32766782 PMC7498326

[25] Li H . Aligning sequence reads, clone sequences and assembly contigs with BWA-MEM, version 2. Preprint, arXiv. 2013. 10.48550/ARXIV.1303.3997

[26] Danecek P, Bonfield JK, Liddle J, et al. Twelve years of SAMtools and BCFtools. *GigaScience* 2021;10:giab008. 10.1093/gigascience/giab008 33590861 PMC7931819

[27] Meyer RS, Curd EE, Schweizer T, et al. The California environmental DNA “CALeDNA” program. Preprint. 2019. 10.1101/503383

[28] Pipes L, Nielsen R. A rapid phylogeny-based method for accurate community profiling of large-scale metabarcoding datasets. *eLife* 2024;13:e85794. 10.7554/eLife.85794 39145536 PMC11377034

[29] Yilmaz P, Parfrey LW, Yarza P, et al. The SILVA and “all-species living tree project (LTP)” taxonomic frameworks. *Nucleic Acids Res* 2014;42:D643–8. 10.1093/nar/gkt1209 24293649 PMC3965112

[30] Edgar RC . MUSCLE: multiple sequence alignment with high accuracy and high throughput. *Nucleic Acids Res* 2004;32:1792–7. 10.1093/nar/gkh340 15034147 PMC390337

[31] Guindon S, Dufayard JF, Lefort V, et al. New algorithms and methods to estimate maximum-likelihood phylogenies: assessing the performance of PhyML 3.0. *Syst Biol* 2010;59:307–21. 10.1093/sysbio/syq010 20525638

[32] Dupuis S, Ojeda V, Gallaher SD, et al. Too dim, too bright, and just right: systems analysis of the *Chlamydomonas* diurnal program under limiting and excess light. *Plant Cell* 2025;37:koaf086. 10.1093/plcell/koaf086 40251989 PMC12136973

[33] Monroe ME, Shaw JL, Daly DS, et al. MASIC: a software program for fast quantitation and flexible visualization of chromatographic profiles from detected LC-MS(/MS) features. *Comput Biol Chem* 2008;32:215–7. 10.1016/j.compbiolchem.2008.02.006 18440872 PMC2487672

[34] Strenkert D, Schmollinger S, Gallaher SD, et al. Multiomics resolution of molecular events during a day in the life of *Chlamydomonas*. *Proc Natl Acad Sci* 2019;116:2374–83. 10.1073/pnas.1815238116 30659148 PMC6369806

[35] Camacho DJ, Perrino C, Merchant SS. Routine operation of a triple quadrupole inductively coupled plasma mass spectrometer (ICP-MS/MS) for determining the elemental composition of various cell types. v1. 2024. 10.17504/protocols.io.eq2lywebevx9/v1

[36] Calatrava V, Tejada-Jimenez M, Sanz-Luque E, et al. *Chlamydomonas reinhardtii*, a reference organism to study algal–microbial interactions: why can’t they be friends? *Plants* 2023;12:788. 10.3390/plants12040788 36840135 PMC9965935

[37] Durán P, Flores-Uribe J, Wippel K, et al. Shared features and reciprocal complementation of the *Chlamydomonas* and *Arabidopsis* microbiota. *Nat Commun* 2022;13:406. 10.1038/s41467-022-28055-8 35058457 PMC8776852

[38] Bai Y, Mori K, Tanaka Y, et al. Isolation and characterization of bacteria from natural microbiota regrown with *Chlamydomonas reinhardtii* in synthetic co-cultures. *Algal Res* 2025;86:103954. 10.1016/j.algal.2025.103954

[39] Glaesener AG, Merchant SS, Blaby-Haas CE. Iron economy in *Chlamydomonas reinhardtii*. *Front Plant Sci* 2013;4:337. 10.3389/fpls.2013.00337 24032036 PMC3759009

[40] Esteves SM, Jadoul A, Iacono F, et al. Natural variation of nutrient homeostasis among laboratory and field strains of *Chlamydomonas reinhardtii*. *J Exp Bot* 2023;74:5198–217. 10.1093/jxb/erad194 37235689

[41] Schmollinger S, Chen S, Strenkert D, et al. Single-cell visualization and quantification of trace metals in *Chlamydomonas* lysosome-related organelles. *Proc Natl Acad Sci* 2021;118:e2026811118. 10.1073/pnas.2026811118 33879572 PMC8072209

[42] Long H, Fang J, Ye L, et al. Structural and functional regulation of *Chlamydomonas* lysosome-related organelles during environmental changes. *Plant Physiol* 2023;192:927–44. 10.1093/plphys/kiad189 36946208 PMC10231462

[43] Dupuis S, Lingappa UF, Purvine SO, et al. Mono-mix strategy enables comparative proteomics of a cross-kingdom microbial symbiosis. *PLoS One* 2026;21:e0340253. 10.1371/journal.pone.0340253 41544079 PMC12810790

[44] Hanschen ER, Marriage TN, Ferris PJ, et al. The *Gonium pectorale* genome demonstrates co-option of cell cycle regulation during the evolution of multicellularity. *Nat Commun* 2016;7:11370. 10.1038/ncomms11370 27102219 PMC4844696

[45] Prochnik SE, Umen J, Nedelcu AM, et al. Genomic analysis of organismal complexity in the multicellular green alga *Volvox carteri*. *Science* 2010;329:223–6. 10.1126/science.1188800 20616280 PMC2993248

[46] de CF, Lemaire SD, Danon A. When unity is strength: the strategies used by *Chlamydomonas* to survive environmental stresses. *Cells* 2019;8:1307. 10.3390/cells8111307 31652831 PMC6912462

[47] de CF, Maes A, Marchand CH, et al. How abiotic stress-induced socialization leads to the formation of massive aggregates in *Chlamydomonas*. *Plant Physiol* 2022;190:1927–40. 10.1093/plphys/kiac321 35775951 PMC9614484

[48] Smith D, Muscatine L, Lewis D. Carbohydrate movement from autotrophs to heterotophs in parasitic and mutualistic symbiosis. *Biol Rev* 1969;44:17–85. 10.1111/j.1469-185X.1969.tb00821.x 4890118

[49] Scott M, Gunderson CW, Mateescu EM, et al. Interdependence of cell growth and gene expression: origins and consequences. *Science* 2010;330:1099–102. 10.1126/science.1192588 21097934

[50] Wang Q, Lin J. Environment-specificity and universality of the microbial growth law. *Commun Biol* 2022;5:891. 10.1038/s42003-022-03815-w 36045217 PMC9433384

[51] Chua ST, Kotar J, Kühl M, et al. Uncoupling growth and division in *Chlamydomonas reinhardtii* colonies: consistent cell cycle regulation under confinement. *ISME Commun* 2025;5:ycaf104. 10.1093/ismeco/ycaf104 40734926 PMC12306439

[52] Rose CJ, Hammerschmidt K. What do we mean by multicellularity? The evolutionary transitions framework provides answers. *Front Ecol Evol* 2021;9:730714. 10.3389/fevo.2021.730714

[53] Grosberg RK, Strathmann RR. The evolution of multicellularity: a minor major transition? *Annu Rev Ecol Evol Syst* 2007;38:621–54. 10.1146/annurev.ecolsys.36.102403.114735

[54] Khona DK, Shirolikar SM, Gawde KK, et al. Characterization of salt stress-induced palmelloids in the green alga, *Chlamydomonas reinhardtii*. *Algal Res* 2016;16:434–48. 10.1016/j.algal.2016.03.035

[55] Lurling M, Beekman W. Palmelloids formation in *Chlamydomonas reinhardtii*: defence against rotifer predators? *Ann Limnol - Int J Limnol* 2006;42:65–72. 10.1051/limn/2006010.

[56] Cheloni G, Slaveykova VI. Morphological plasticity in *Chlamydomonas reinhardtii* and acclimation to micropollutant stress. *Aquat Toxicol* 2021;231:105711. 10.1016/j.aquatox.2020.105711 33338702

[57] Brenner D.J., Krieg N.R., Staley J.T. (eds.). Bergey’s Manual® of Systematic Bacteriology. Boston, MA: Springer US, 2005, 10.1007/0-387-29298-5.

[58] Gould AL, Osland HK. Strain-level variation in microbial symbiosis: lessons from the *Siphamia–Photobacterium mandapamensis* system. *Trends Microbiol* 2025;33:580–2. 10.1016/j.tim.2025.02.010 40107953

[59] Flemming HC, Wingender J, Szewzyk U, et al. Biofilms: an emergent form of bacterial life. *Nat Rev Microbiol* 2016;14:563–75. 10.1038/nrmicro.2016.94 27510863

[60] Flemming HC, Wuertz S. Bacteria and archaea on earth and their abundance in biofilms. *Nat Rev Microbiol* 2019;17:247–60. 10.1038/s41579-019-0158-9 30760902

