# Supplementary material for: Field strains of the unicellular alga Chlamydomonas reinhardtii exhibit multicellular characteristics that shape their interactions
Source: ISME J. 2026 Jul 28;20(1):wrag202. doi: 10.1093/ismejo/wrag202 (PMC13525550; doi:10.1093/ismejo/wrag202)
Supplement: Supplementary file 1 [file supplementary_material_wrag202.zip › SupplementalFigures_revised2_wrag202.pdf]

Supplemental figures

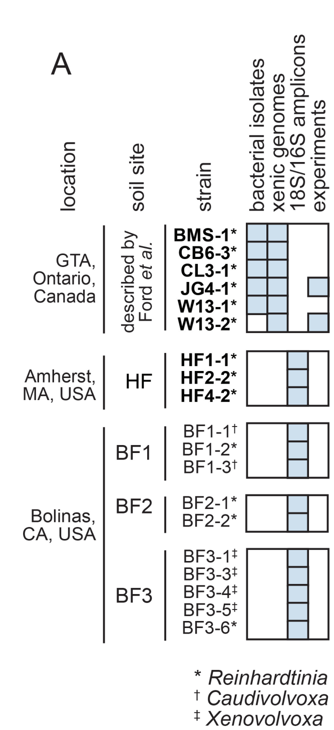

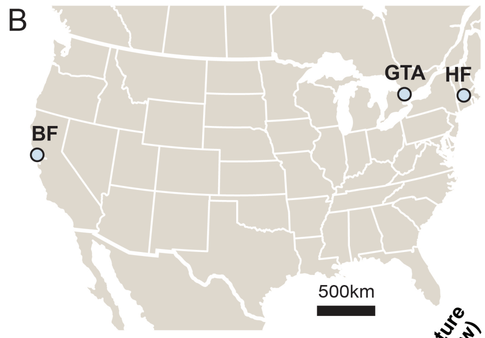

C

site	location	GPS (N, W)	sample date	temperature (high, low)
HF	Hampshire College Farm, Amherst, MA	42.33017, -72.5286	10 June, 2023	33.5°C, -23°C
BF1	Paradise Valley Farm, Bolinas, CA	37.92151, -122.701	13 July, 2023	33°C, 3°C
BF2	Paradise Valley Farm, Bolinas, CA	37.92239, -122.701	13 July, 2023	33°C, 3°C
BF3	Paradise Valley Farm, Bolinas, CA	37.92185, -122.702	13 July, 2023	33°C, 3°C

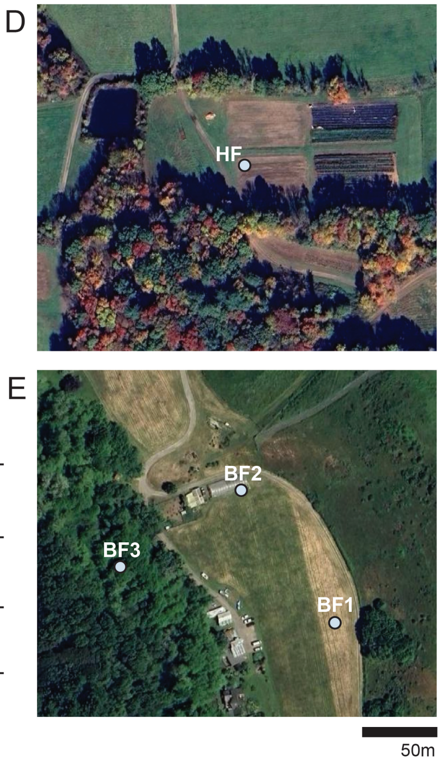

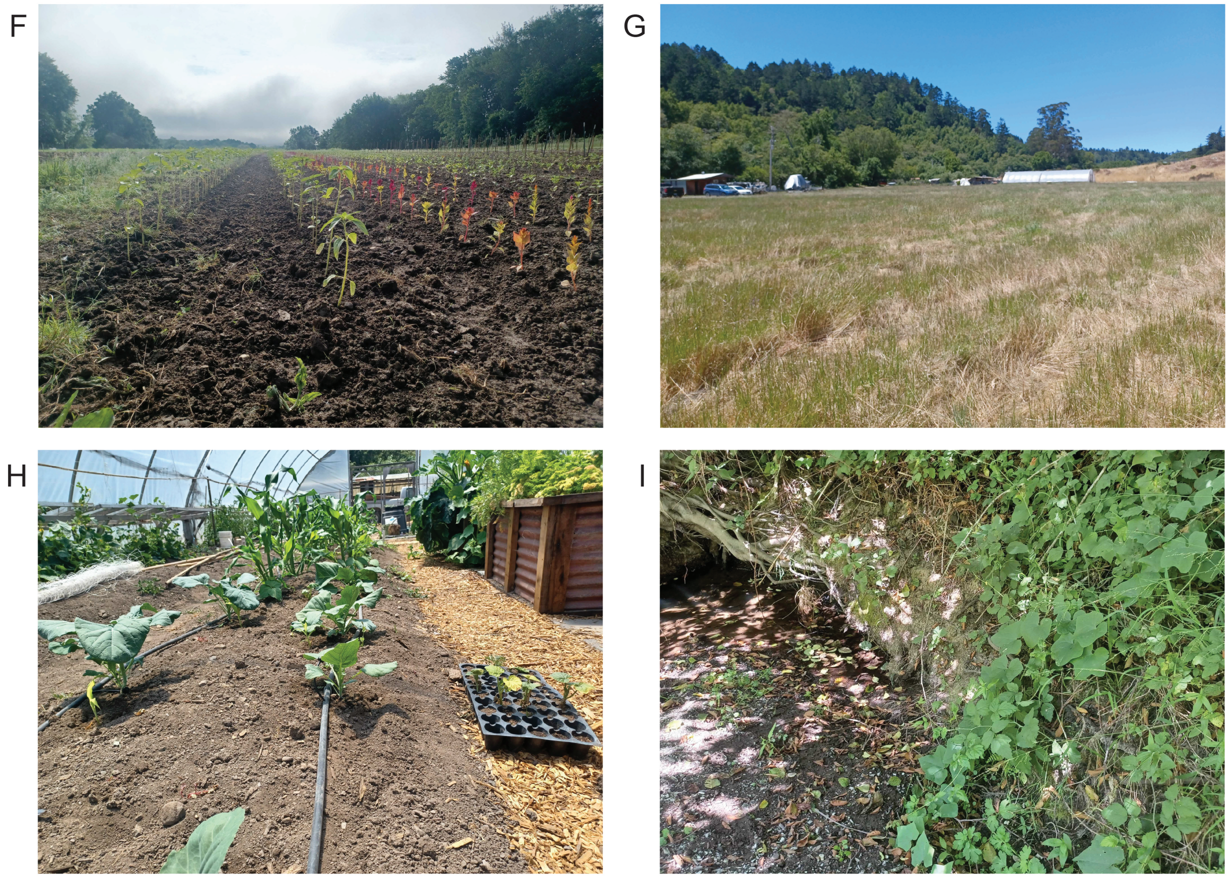

Figure S1. Description of field sites and field strains used in this study. ***A***, List of algal strains from each field site. Bolded strain names indicate members of the species *C. reinhardtii*; symbols indicate broader taxonomic groups. Shaded boxes indicate which components of this study each strain was involved in. ***B***, Map of North America indicating locations of field sites. ***C***, Field site metadata. ***D-E***, Satellite images of Hampshire College Farm (***D***) and Paradise Valley Farm (***E***), indicating sample sites. ***F-I***, Context photos of sample sites HF, an active farm field (**F**), BF1, an empty farm field (**G**), BF2, an active farm field hoop house (**H**), and BF3, a stream bed adjacent to the farm (**I**).

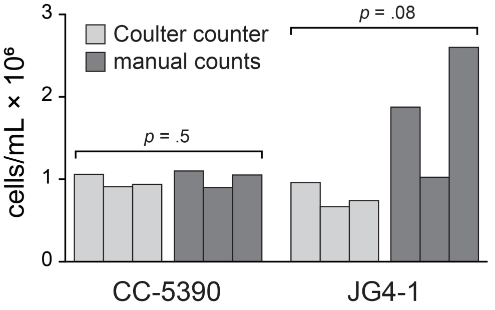

Figure S2. Comparison of cell counts obtained by Coulter counter vs. manually in a hemocytometer, illustrated with triplicate cultures of lab strain CC-5390 and field strain JG4-1 counted both ways. For the lab strains, the different methods yielded very similar results. Therefore, we were comfortable using Coulter counter data. For the field strains, they were less similar. Therefore, we favored the manual counts.

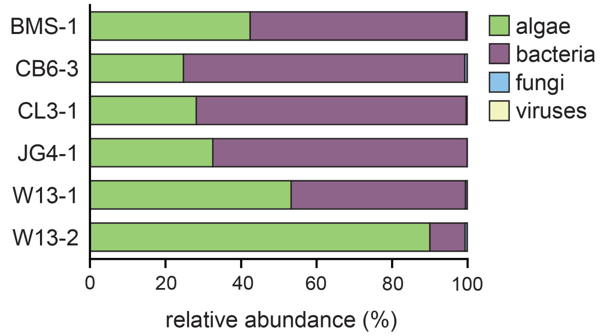

Figure S3. Taxonomic composition of genome sequence reads from xenic *C. reinhardtii* strains. We note that this analysis is biased by the sequences represented in the reference database, and therefore almost certainly underestimates the abundance of viruses (especially RNA viruses).

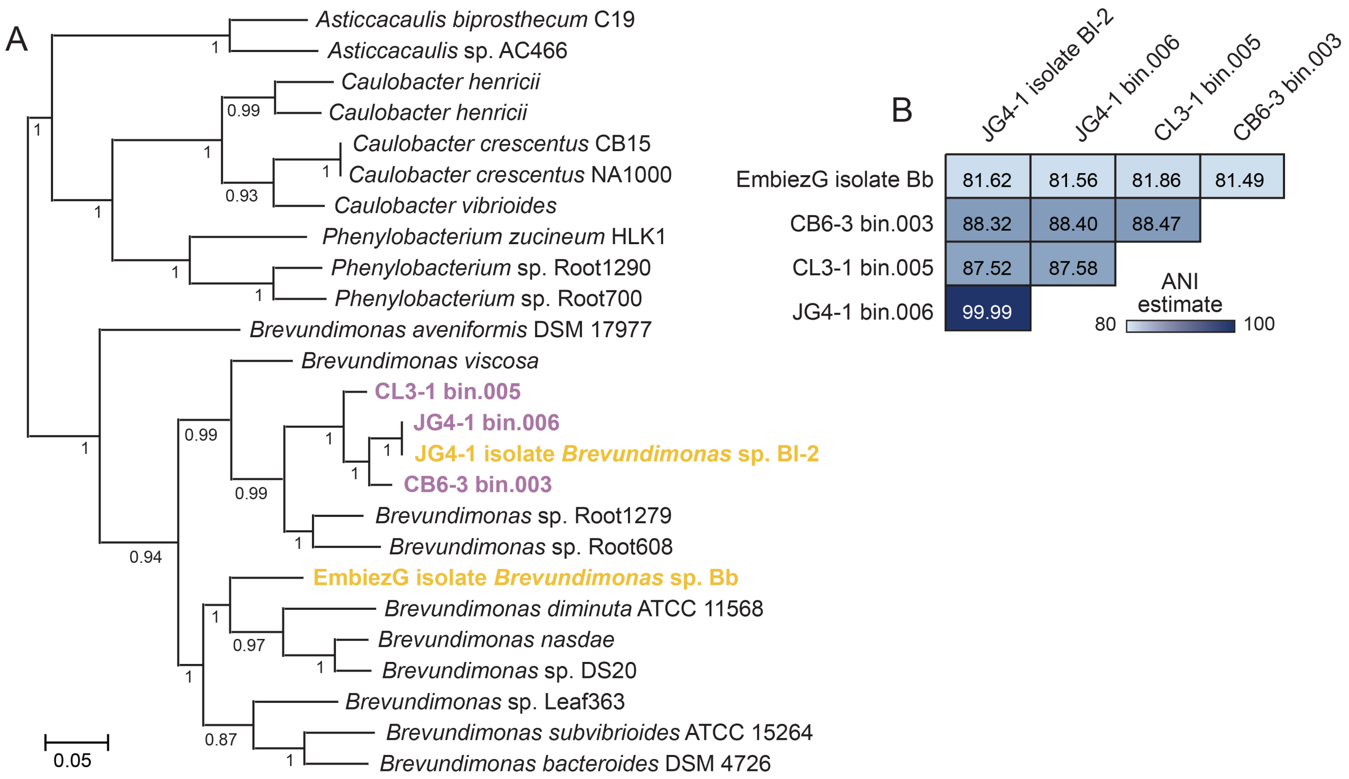

Figure S4. Genomic comparisons of Brevundimonads associated with *C. reinhardtii* field strains.

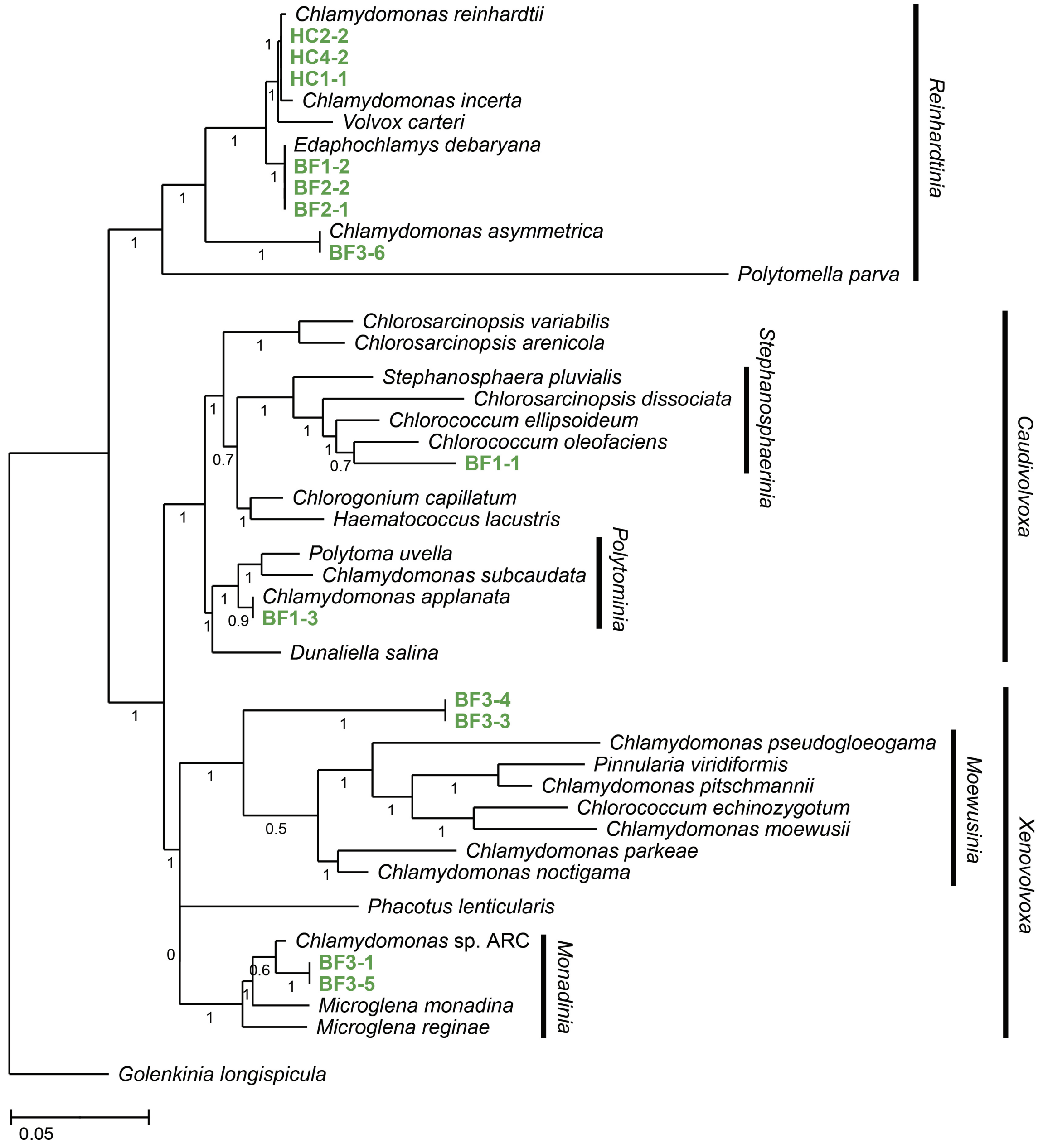

Figure S5. 18S tree showing the positions of algal strains enriched from Amherst and Bolinas within the order Volvocales.

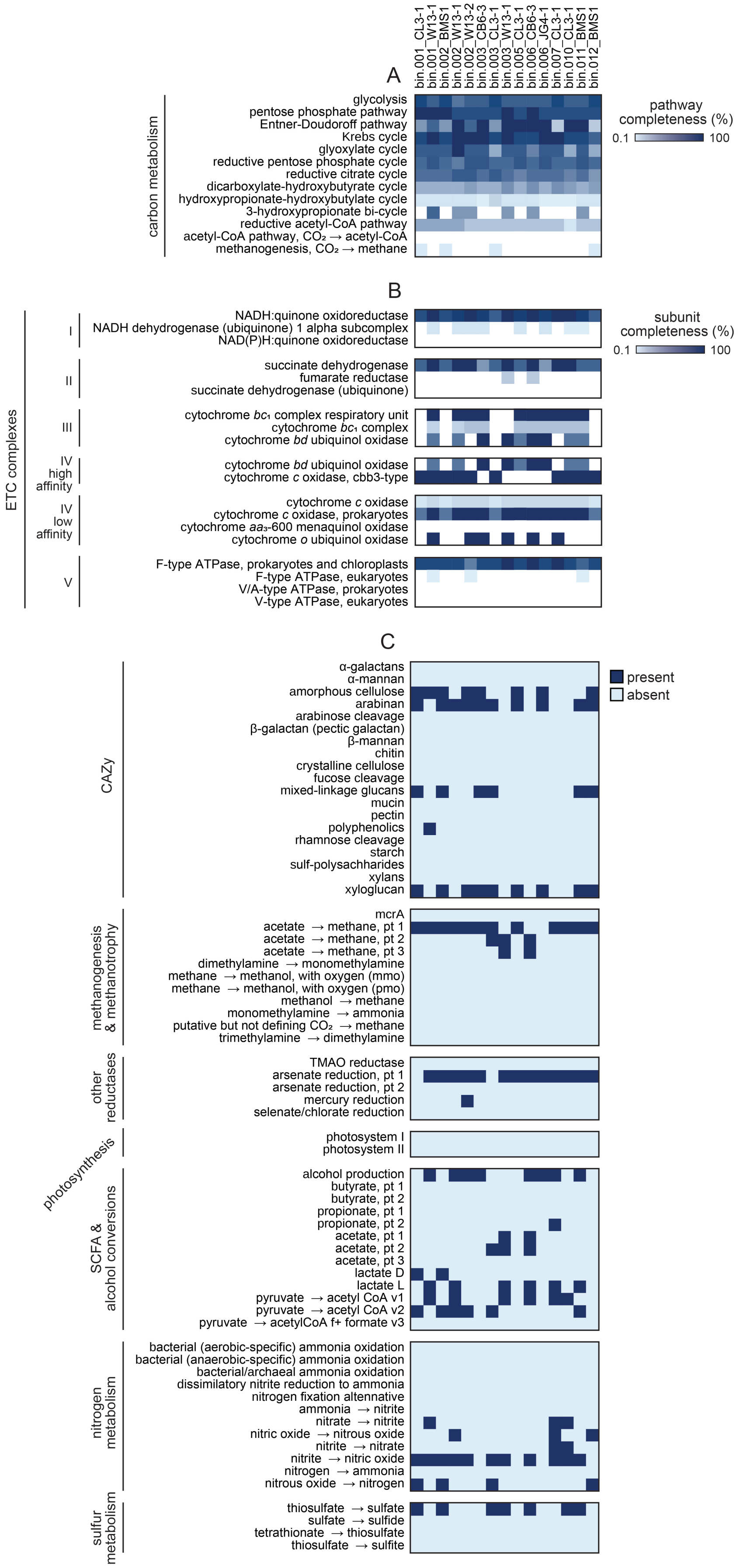

Figure S6. DRAM product summaries of MAGs representing *C. reinhardtii* associated bacteria, indicating carbon metabolism pathway completeness (***A***), electron transport chain subunit completeness (***B***), and presence of specific functions (***C***).

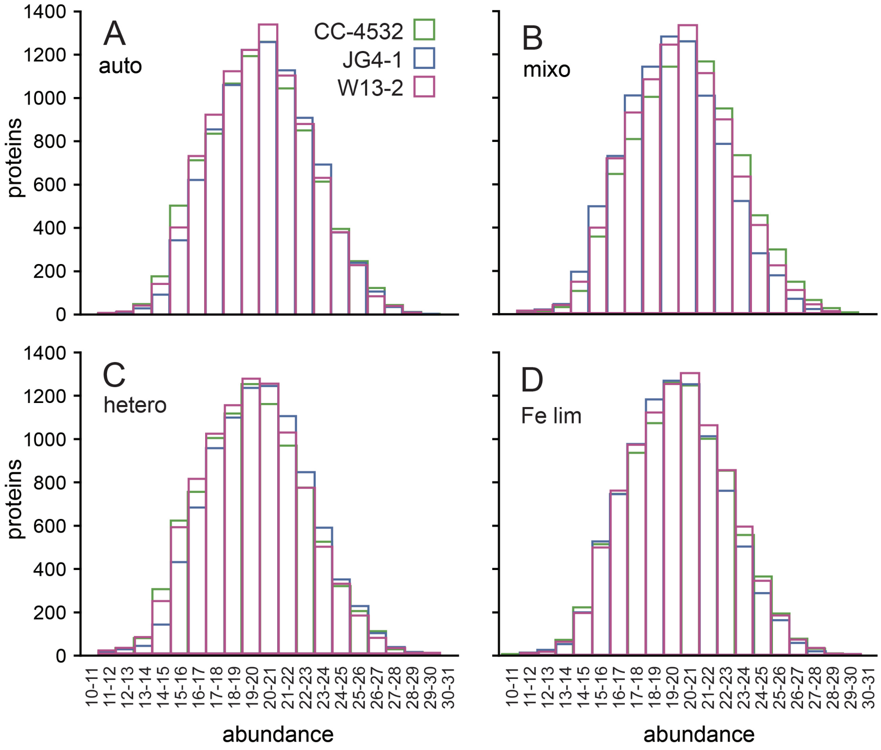

Figure S7. Strain-level differences did not confound protein detection, illustrated by overlain histograms of protein abundance distributions recovered under autotrophic (***A***), mixotrophic (***B***), heterotrophic (***C***), and mixotrophic Fe limited (***D***) growth.

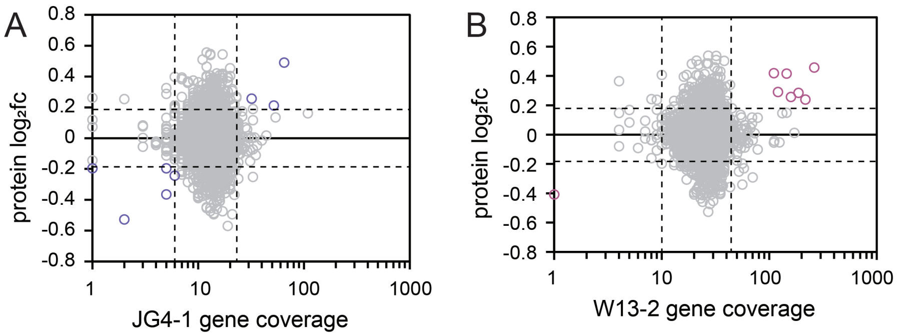

C

	gene ID	symbol	defline
JG4-1	Cre12.g485350		
	Cre11.g467730		[histone H3]-lysine(4) N-trimethyltransferase
	Cre13.g565321		Adenylosuccinate lyase
W13-2	Cre07.g329950		
	Cre04.g231418		Protein kinase-like domain
	Cre03.g150900		P-loop containing nucleoside triphosphate hydrolase
	Cre12.g541950		Antifreeze protein, type I
	Cre12.g542000		D-tyrosyl-tRNA(Tyr) deacylase
	Cre11.g467730		[histone H3]-lysine(4) N-trimethyltransferase
	Cre07.g354250	PGK2	Phosphoglycerate kinase

D

	gene ID	symbol	defline
JG4-1	Cre04.g214250	AGO2	Argonaute-like protein
	Cre16.g658000		Putative metallochaperone
	Cre13.g566200	CGL112	Possible transcription factor, conserved in the green lineage
	Cre17.g745297		
	Cre01.g020701		
W13-2	Cre01.g019400		

Figure S8. A small subset of proteins with significantly higher or lower protein abundances also exhibited higher or lower gene coverage. ***A*-*B***, Coverage distributions of JG4-1 (***A***) and W13-2 (***B***) genome sequence reads mapped onto the CC-4532 reference genome, plotted against average log_2 fold change of protein abundance under mixotrophy. Dashed lines indicate 2 standard deviations from the mean. ***C***, Proteins with significantly higher gene coverage and protein abundance. ***D***, Proteins with significantly lower gene coverage and protein abundances.

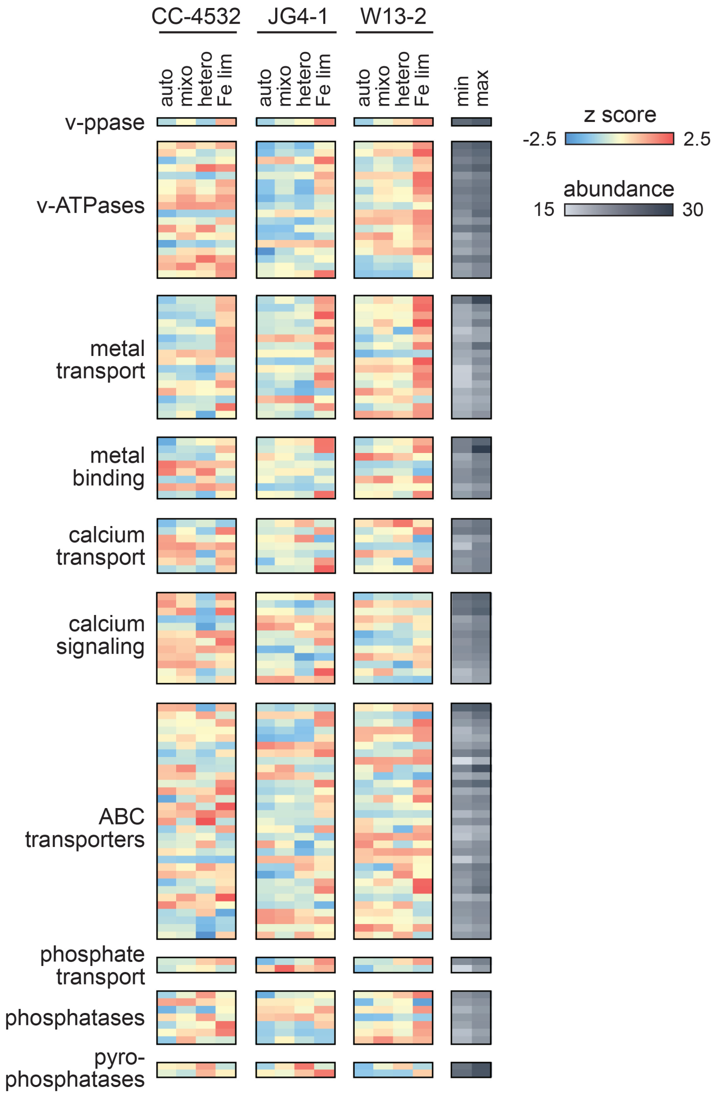

Figure S9. Normalized abundance (z-scores of mean abundance values) and abundance range (minimum and maximum abundance values) of candidate acidocalcisomal proteins identified by Long *et al* [42].
